# Effect of laryngeal mask airway versus endotracheal intubation on survival in out-of-hospital cardiac arrest: a meta-analysis of randomized controlled trials

**DOI:** 10.3389/fpubh.2026.1877439

**Published:** 2026-08-17

**Authors:** Dongxia Zhang, Shuang Li, Zhaofan Mo, Huazhen Xie, Yihui Lin, Quanle Liu, Ruifeng Zeng, Danwen Zheng, Xiaotu Xi, Zunjiang Li, Banghan Ding

**Affiliations:** 1 The Second Clinical College of Guangzhou University of Chinese Medicine, Guangzhou, China; 2 The First Clinical College of Guangzhou University of Chinese Medicine, Guangzhou, China; 3 The Second Affiliated Hospital of Guangzhou University of Chinese Medicine, Guangzhou, China

**Keywords:** airway management, endotracheal intubation, laryngeal mask airway, meta-analysis, out-of-hospital cardiac arrest, systematic review

## Abstract

**Objective:**

To compare the effectiveness and safety of laryngeal mask airway (LMA) versus endotracheal intubation (ETI) on resuscitation outcomes in patients with out-of-hospital cardiac arrest (OHCA).

**Methods:**

Randomized controlled trials (RCTs) were searched in PubMed, Embase, the Cochrane Library, Web of Science, CNKI, Wanfang, VIP, and CBM from database inception to May 31, 2025. The methodological quality and risk of bias of the included RCTs were evaluated with the Cochrane ROB2 tool. We employed RevMan 5.3 and TSA software for all statistical analyses, including meta-analysis, sensitivity analysis, and trial sequential analysis (TSA). The certainty of the evidence was graded according to the GRADE approach. The primary outcomes included airway establishment time and CPR success rate, and the secondary outcomes included first-attempt intubation success rate, mortality, survival rate, left ventricular ejection fraction, and adverse events.

**Results:**

Nineteen RCTs (11,982 patients) were included. Compared with ETI, LMA was associated with a significantly shorter airway establishment time (MD: −86.95 s; 95% CI: −103.00, −70.90; *p* < 0.00001), higher first-attempt success rate (RR: 1.44; 95% CI: 1.32, 1.58; *p* < 0.00001), improved CPR success rate (RR: 1.44; 95% CI: 1.25, 1.67; *p* < 0.00001), and LVEF (SMD: 1.57; 95% CI: 0.65, 2.48; *p* < 0.00001). No significant differences were observed in survival rate (RR: 0.98; 95% CI: 0.88, 1.09; *p* = 0.69), nor mortality (RR: 1.00; 95% CI: 0.99, 1.01; *p* = 0.69). LMA was associated with a lower rate of vocal cord injury (RR: 0.11; 95% CI: 0.03, 0.47; *p* = 0.003), although this finding is based on a small number of events and should be interpreted cautiously. The quality of the GRADE evidence ranged from very low to high.

**Conclusion:**

Compared with ETI, LMA demonstrates significantly higher operational efficiency and a favorable safety profile in the management of OHCA. However, given that survival and mortality outcomes are comparable between the two strategies, LMA may be considered a reasonable alternative to ETI, particularly when minimizing interruptions in chest compressions is a priority or when ETI expertise is limited. The choice of airway device should be individualized rather than uniformly prioritized. In summary, LMA may offer procedural advantages and fewer airway-related complications, while evidence for a survival benefit remains insufficient.

**Systematic review registration:**

https://www.crd.york.ac.uk/prospero/display_record.php?ID=CRD42025645299, identifier (CRD42025645299).

## 1 Introduction

Out-of-hospital cardiac arrest (OHCA) is defined as an unexpected cessation of cardiac mechanical activity and blood circulation that occurs outside of the hospital setting (1, 2). It is estimated that OHCA has a global incidence among adults of approximately 55 per 100,000, while the overall survival rate typically ranges from 10 to 20% (3). Survivors of OHCA frequently experience significant neurological morbidity, with studies indicating that long-term cognitive impairment or functional dependency affects up to 50% of this population. This substantial burden highlights the critical need to optimize resuscitation strategies (4, 5). Immediate initiation of cardiopulmonary resuscitation (CPR) and advanced airway management are fundamental components of the treatment algorithm for OHCA (6, 7). Endotracheal intubation (ETI) is traditionally regarded as the gold standard for managing airways (8). However, ETI is associated with several challenges, such as its technical complexity, significant time requirements, and the risk of periprocedural complications including esophageal intubation and airway trauma (9). In contrast, a growing body of evidence suggests that the laryngeal mask airway (LMA) represents a promising alternative for airway management in OHCA, offering several potential advantages (10). The LMA is a supraglottic device utilized for securing an airway during resuscitation or anesthesia. Evidence indicates that LMA insertion is associated with a higher first-attempt success rate and a reduced time to ventilation (11), thereby minimizing interruptions in chest compressions and helping to maintain continuous cerebral perfusion during CPR. Compared to ETI, the design of LMA is associated with reduced oropharyngeal trauma, mitigating the risk of complications such as bleeding, aspiration, and hemodynamic instability (12). Despite accumulating evidence supporting the efficacy of LMA, current studies show inconsistent findings regarding its comparative effectiveness relative to ETI—particularly in terms of survival and neurological outcomes in patients with OHCA—which may be attributed to a lack of comprehensive systematic reviews focused on this population. Consequently, given the expanding body of literature, there is a compelling need to rigorously evaluate and compare the clinical efficacy and safety of LMA versus ETI in OHCA using updated and optimized methodologies based on the Preferred Reporting Items for Systematic Reviews and Meta-Analyses (PRISMA) guidelines.

Our systematic review and meta-analysis overcome key limitations present in earlier meta-analyses, including one Chinese-language study published in 2018 (13) and another from 2019 (14). Both of these previous analyses incorporated a limited number of studies (only 9 and 13, respectively) and, crucially, combined data from both randomized controlled trials (RCTs) and non-randomized studies, such as observational cohort studies. This methodological heterogeneity contributed to their inconsistent outcomes. The 2018 publication (13) reported that ETI was associated with significantly better outcomes for return of spontaneous circulation (ROSC) (OR 1.51) and one-month survival (OR 1.16). Similarly, the 2019 study (14) concluded that ETI was superior to LMA in terms of ROSC (RR = 0.72), survival to admission (RR = 0.85), and survival to discharge (RR = 0.90). However, these conclusions were based on relatively small and heterogeneous cohorts—often comprising observational or retrospective studies—with limited methodological rigor and without strict adherence to the Preferred Reporting Items for Systematic Reviews and Meta-Analyses (PRISMA) guidelines.

Therefore, this systematic review and meta-analysis aims to provide a more robust and updated comparison of the clinical efficacy and safety of LMA versus ETI in patients with OHCA, by synthesizing evidence exclusively from randomized controlled trials.

To our knowledge, this study represents the first comprehensive meta-analysis on this topic that adheres strictly to an RCT-only inclusion criterion, thereby eliminating the methodological heterogeneity introduced by observational studies that confounded earlier syntheses. Furthermore, our analysis incorporates several recently published RCTs not included in any prior pooled analysis, updates the evidence base to 2025, and applies rigorous sensitivity analyses—including pre-specified analyses excluding the largest trial (AIRWAYS-2) for the three outcomes to which it contributed, in order to transparently assess weight imbalance—as well as trial sequential analysis (TSA) and GRADE certainty ratings. These methodological enhancements distinguish the present work from previous meta-analyses and provide a more reliable quantitative foundation for clinical decision-making in prehospital airway management.

## 2 Data and methods

### 2.1 Literature databases for search

Without language or geographic restrictions, a comprehensive retrieval of the bibliography database, including Embase, the Cochrane Library, PubMed, the Web of Science, the China Science and Technology Journal Database (VIP), the Wanfang Database, China Biology Medicine disc (CBM), and China National Knowledge Infrastructure (CNKI), was conducted from inception to May 31, 2025 to screen eligible studies.

### 2.2 PICOS criteria for screening studies

All the studies consistent with the following PICOS terms were included. ① Participants (P): Individuals who were diagnosed with OHCA based on the absence of signs of circulation, unresponsiveness, and apnea (consistent with Utstein-style criteria) and were aged more than 18 years. ② Interventions (I and C): In the control group, individuals with OHCA received ETI, whereas OHCA patients in the experimental group were given LMA. ③ Outcomes: The primary outcomes included the airway establishment time and success rate of CPR; the secondary outcomes included the success rate of one-time intubation, mortality, survival rate and LVEF. Moreover, adverse events were also assessed to compare safety. To ensure uniformity and transparency, operational definitions were applied for outcome extraction: airway establishment time was defined as the time from the start of airway management to confirmation of successful device placement, expressed in seconds; CPR success rate was defined as return of spontaneous circulation (ROSC) at any time during resuscitation, regardless of duration or subsequent outcome, and when studies reported multiple time points we used the definition provided as the primary endpoint; first-attempt intubation success rate was successful placement of the assigned airway device on the first insertion attempt per the study’s own criteria; mortality was all-cause death before hospital discharge, with 30-day mortality accepted as a proxy when discharge data were unavailable, and sensitivity analyses confirmed that this choice did not influence the pooled estimate; survival rate was survival to hospital discharge, and where 30-day survival or neurologically intact survival was reported as the primary endpoint we extracted survival-to-discharge data if available, otherwise the study’s primary survival definition was accepted and noted; LVEF was left ventricular ejection fraction measured by echocardiography after ROSC, typically within the first 24 h post-resuscitation; adverse events were recorded as defined by each individual study, with no additional standardization beyond the categories listed in our subgroup analysis. For each outcome, we recorded the exact definition, time point, and measurement method used in the original study, and when definitions varied across studies, these differences were documented and considered in the interpretation of results. ④ Studies: Both parallel-group and cluster-randomized controlled trials were eligible. For cluster-randomized trials, the study was included only if the original analysis had appropriately accounted for the clustered design (e.g., using mixed-effects models or generalized estimating equations) and the reported effect estimate with its standard error could be directly pooled with individually-randomized trials without further adjustment. Two included studies—Lee et al. (15) and Benger et al. (16)—employed a cluster-randomized design and met this criterion, thus no additional unit-of-analysis correction was required in our meta-analysis.

### 2.3 Qualifications for exclusion

① Duplicate or overlapping publications; ② unavailability of the full text despite exhaustive retrieval efforts; ③ studies in which co-interventions (e.g., specific medications like advanced epinephrine protocols, or additional devices like mechanical CPR) were administered disproportionately between the LMA and ETI groups, potentially confounding the effect of the airway intervention; and ④ redundant publications with overlapping authorship and outcomes.

### 2.4 Search strategy

The search strategy was developed by two reviewers (DX Zhang and ZF Mo). MeSH phrases from the PICOS, comprising P + I + C + O + S or P + I + O + S, were combined to form our retrieval method. We searched according to P + I + C when the number of articles was small at the search stage and then manually screened them on the basis of the inclusion and exclusion criteria. The complete search strategies for all eight databases, including the exact search terms, Boolean operators, and the date of the last search (from inception to May 31, 2025), are presented in a unified Supplementary file to ensure full reproducibility.

### 2.5 Data collection and evaluation

#### 2.5.1 Overview

This systematic review and meta-analysis was conducted in accordance with the Preferred Reporting Items for Systematic Reviews and Meta-Analyses (PRISMA) guidelines [ref]. A total of 19 randomized controlled trials (RCTs) comprising 11,982 patients were included, representing a substantial expansion in sample size and statistical power compared with prior meta-analyses. To ensure methodological rigor and transparency, we employed several advanced analytical approaches: (1) trial sequential analysis (TSA) to control for type I and II errors and to assess the conclusiveness of the evidence; (2) the updated Cochrane Risk of Bias 2 (RoB2) tool for comprehensive risk-of-bias assessment; and (3) the Grading of Recommendations, Assessment, Development, and Evaluations (GRADE) framework to evaluate the certainty of evidence for each outcome. In addition, we explicitly accounted for key confounders often neglected in previous studies, including geographic variability and differences in provider skill level (e.g., paramedic- versus physician-performed intubation). The following sections detail the search strategy, eligibility criteria, data extraction procedures, and statistical methods employed in this study.

#### 2.5.2 Selection of trials

**Figure 1:**
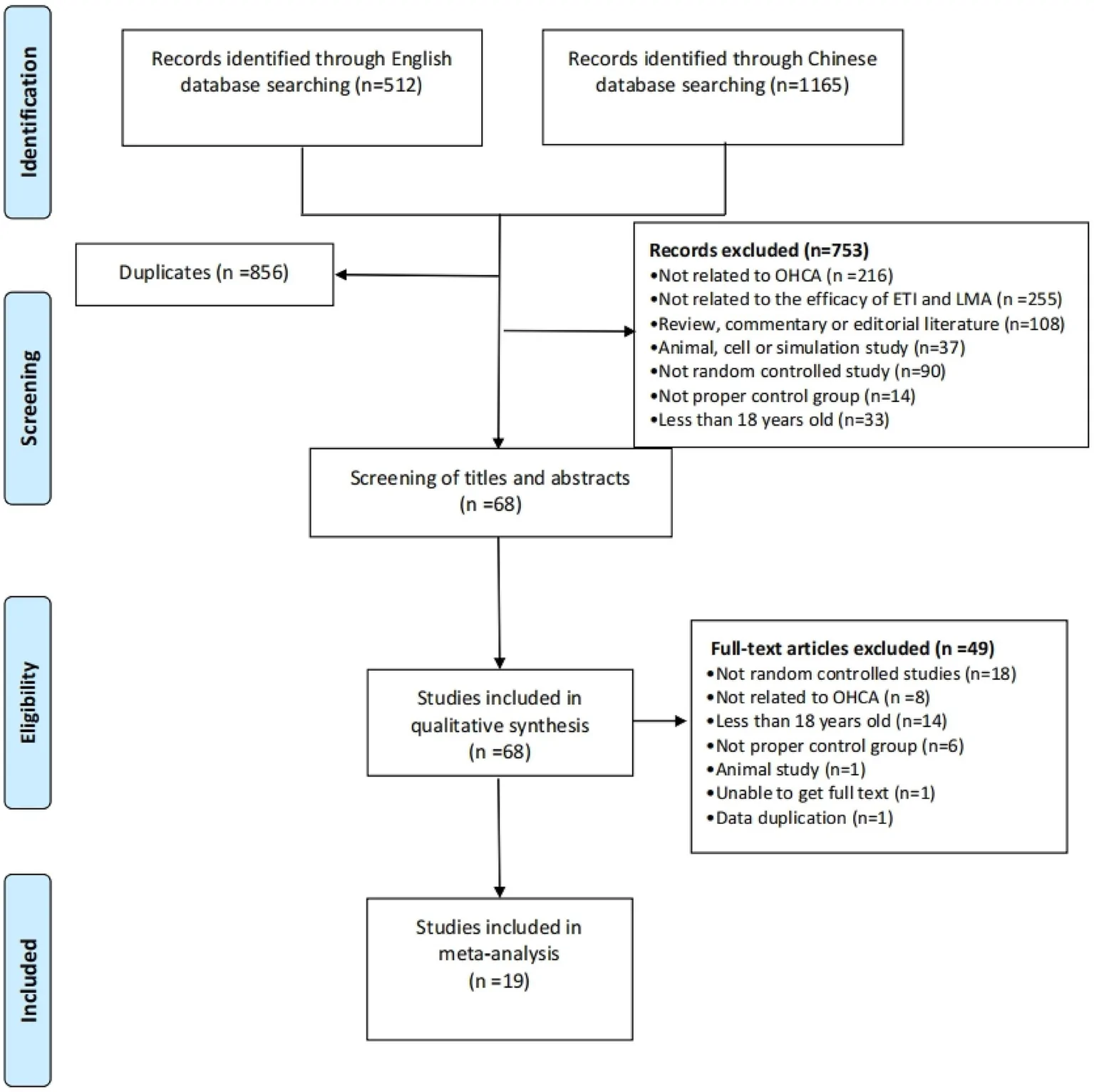
PRISMA flow chart of the study selection process in the systematic review.

All retrieved records were imported into NoteExpress (version: 4.0.0.9855) for management and deduplication. The selection process was conducted in two stages. First, two reviewers (DX Zhang and ZF Mo) independently screened titles and abstracts of all unique records to identify potentially eligible studies. Second, the full texts of these potentially eligible articles were retrieved and independently assessed for final inclusion by two additional reviewers (S Li and HZ Xie). Any discrepancies at either stage were resolved through consensus among all authors. If consensus could not be reached, a third reviewer (BH Ding) was consulted. The inter-rater agreement for study selection, measured by Cohen’s kappa coefficient, was 0.86, indicating almost perfect agreement. The reasons for full-text exclusion were documented and are presented in a PRISMA flow diagram (Figure 1).

#### 2.5.3 Data extraction and management

A standardized data extraction form was piloted and refined in Microsoft Excel (2019 version). Two reviewers (ZJ Li and YH Lin) independently extracted data from each included study. Discrepancies were cross-checked and resolved through discussion, with involvement of a third reviewer (BH Ding) when necessary. Extracted information included: first author, year of publication, country, study design, sample size, patient characteristics (age, sex), details of the LMA and ETI interventions, co-interventions during CPR, and outcome data as specified in Section 2.2. For each outcome, we also extracted the definition used by the study (e.g., ROSC timing, mortality follow-up period, survival endpoint) and the method of measurement, to allow assessment of definitional consistency across trials. For studies with multiple publications, the most comprehensive or up-to-date data were used.

For missing or unclear data, we contacted the corresponding authors of the primary studies via email up to two times over a four-week period. If no response was received, the data were considered unavailable, and the study was included only for outcomes with available data. This approach was documented in the risk of bias assessment under the ‘missing outcome data’ domain.

#### 2.5.4 Risk of bias (ROB) evaluation

Two reviewers (DX Zhang and DW Zheng) independently evaluated the risk of bias (ROB) in the included studies using the RoB2 tool, in accordance with the Cochrane Handbook version 6.3. Key domains assessed included the randomization process, deviations from intended interventions, missing outcome data, outcome measurement, and selection of the reported result. For each domain, reviewers assigned a judgment of “low risk of bias,” “some concerns,” or “high risk of bias” based on the tool’s signaling questions. The overall risk of bias for each study was then derived algorithmically as follows: an overall judgment of “low risk of bias” required all domains to be judged as low risk; an overall judgment of “some concerns” was assigned if the study had at least one domain with some concerns but no domain judged as high risk; and an overall judgment of “high risk of bias” was assigned if the study had at least one domain judged as high risk or if it had multiple domains with some concerns that significantly undermined confidence in the results, in accordance with the RoB2 guidance (17). Any discrepancies between reviewers were resolved through consensus. If consensus was not reached, a third reviewer (XT Xi) was consulted.

#### 2.5.5 Meta-analysis

For continuous variables, pooled estimates were calculated as mean differences (MDs) with 95% confidence intervals (CIs) when the same measurement scale was used across studies. When different scales were used to measure the same construct, or when data were presented in formats requiring transformation, standardized mean differences (SMDs) with 95% CIs were calculated using Review Manager (RevMan version 5.3, The Cochrane Collaboration). For dichotomous variables, pooled estimates were calculated as relative risks (RRs) with 95% CIs. Two included trials were cluster-randomized (15, 16). For these studies, we extracted the effect estimates and standard errors that had already been adjusted for clustering by the original authors. Consequently, no unit-of-analysis error was introduced, and these adjusted estimates were directly pooled with those from parallel-group trials. A sensitivity analysis excluding the largest cluster-randomized trial (16) confirmed that its inclusion did not materially alter the pooled results. Lee et al. (15), the other cluster-randomized trial, contributed a smaller number of participants and did not dominate any analysis; its exclusion was covered within the leave-one-out sensitivity analyses, which also did not alter the conclusions.

Given the anticipated clinical and methodological diversity across studies (e.g., variations in patient populations, provider skill levels, and healthcare settings), we chose *a priori* to apply a random-effects model using the DerSimonian and Laird method as the primary analytic approach for all outcomes. This model assumes that the true treatment effect may vary across studies and provides a more conservative estimate by incorporating both within-study and between-study variance. However, recognizing that random-effects models can produce imprecise estimates when heterogeneity is negligible, we pre-specified the following rule: if the I^2^ statistic was less than 50% and the Cochran’s Q test (reported as the Chi^2^ statistic in RevMan) was non-significant (*p* > 0.10), a fixed-effect model was deemed appropriate and was applied; otherwise, the random-effects model was retained. This rule was applied consistently to all primary and secondary outcomes, as well as to sensitivity analyses in which heterogeneity decreased below the 50% threshold, such as the sensitivity analysis for first-attempt intubation success rate.

Heterogeneity was quantified using the I^2^ statistic and interpreted as follows: 0–25% (little), 25–50% (low), 50–75% (moderate), and >75% (high heterogeneity) (18–20). In accordance with our pre-specified rule, the I^2^ statistic and Cochran Q test were used to guide model selection, with the fixed-effect model applied only when both criteria (I^2^ < 50% and Q *p* > 0.10) were met. All statistical tests were two-sided, and a *p*-value < 0.05 was considered statistically significant, except for the heterogeneity test where *p* < 0.1 was used.

#### 2.5.6 Subgroup analysis

To provide a comprehensive safety profile of LMA versus ETI, we planned a subgroup analysis based on the type of adverse events. Specifically, for studies reporting adverse event data, we categorized events into clinically meaningful subtypes, including but not limited to: total adverse events, vocal cord damage, tooth loss, mucous membrane damage, and aspiration of gastric contents. Pooled estimates (RRs with 95% CIs) were calculated separately for each adverse event subtype using the meta-analytic models described in Section 2.5.4. This approach allowed for a detailed comparison of specific risks associated with each airway management strategy.

#### 2.5.7 Sensitivity analysis

Sensitivity analysis was performed to assess the robustness of the pooled results (21). To evaluate the influence of individual studies, we employed a leave-one-out method, in which the meta-analysis was sequentially repeated after excluding one study at a time to examine its impact on the pooled effect estimates and heterogeneity levels. Additionally, we conducted sensitivity analyses based on study-level characteristics that could potentially bias the overall estimates, including: (1) methodological quality, by excluding studies with an overall judgment of ‘high risk of bias’ from the RoB2 assessment; (2) sample size, by excluding small studies with fewer than 100 participants; and (3) statistical model choice, by applying a fixed-effect model to assess whether the choice of statistical model influenced the conclusions. A sensitivity analysis was also performed to investigate the impact of potential outliers, defined as studies whose 95% confidence intervals did not overlap with the 95% confidence interval of the pooled estimate (22).

#### 2.5.8 Trial sequential analysis (TSA)

TSA was conducted for key outcomes including survival rate, mortality, first-attempt intubation success rate, CPR success rate, time to airway establishment, and incidence rate to control type I and type II error rates. Using TSA software (v. 0.9.5.10 beta, Copenhagen Trial Unit), confidence intervals (CIs) were adjusted for sparse data, and cumulative meta-analysis was re-evaluated. Definitive conclusions were considered established if the cumulative Z-curve crossed either the trial sequential monitoring boundary or the required information size (RIS) threshold, indicating that no further studies are needed. The TSA was configured with a model variance-based diversity (D^2^) correction, 80% power and a two-sided 5% risk of type I error (23).

#### 2.5.9 Confidence in the proof

The overall certainty of evidence for each pooled outcome from the meta-analysis was evaluated using the GRADEpro tool (GRADEpro GDT, available at https://www.gradepro.org/) by two reviewers (QL Liu and RF Zeng) independently. The evidence was initially classified as high quality and was subsequently downgraded based on assessments of risk of bias, inconsistency, indirectness, imprecision, and publication bias. For the risk of bias domain, we downgraded the evidence if a substantial proportion of the pooled studies (typically >25% of the weight in the meta-analysis) came from studies judged to be at high risk of bias. Conversely, we considered upgrading the evidence when a large effect size (RR > 2 or <0.5) or a very large effect (RR > 5 or <0.2) was observed, in accordance with GRADE guidance. Each upgrade was rated as “strong association” (+1) or “very strong association” (+2) based on the magnitude and consistency of the effect. The net rating after all downgrades and upgrades determined the final certainty of evidence. The rationale for downgrading was documented for each outcome. Disagreements were resolved through consensus. The certainty of evidence was categorized into four levels: high, moderate, low, or very low, and the results are presented in a ‘Summary of Findings’ table (Table 1).

**Table 1:** Summary findings of the grading recommendation assessment, development, and evaluation (GRADE) methods.

Certainty assessment							Summary of findings				Comments
Participants (studies) follow-up	Risk of bias	Inconsistency	Indirectness	Imprecision	Other consideration	Overall certainty of evidence	Events		Anticipated relative effect (95% CI)	Anticipated absolute effects (95% CI)	
							Control	Experiment			
One-time intubation success rate 7,964 (16 RCTs)	Very serious^a^	Not serious	Not serious	Not serious	Very strong association^d^	⨁⨁⨁⨁ High	2297/3354 (68.5%)	4005/4610 (86.9%)	RR 1.25 (1.22 to 1.28)	171 more per 1,000 (from 151 more to 192 more)	Risk of bias (−2^a^), Upgraded: very strong association (+2^d^)
Success rate of cardiopulmonary resuscitation 1,185 (13 RCTs)	Very serious^a^	Not serious	Not serious	Not serious	Strong association^e^	⨁⨁⨁◯ Moderate	153/600 (25.5%)	217/585 (37.1%)	RR 1.44 (1.25 to 1.67)	112 more per 1,000 (from 64 more to 171 more)	Risk of bias (−2^a^), Upgraded: strong association (+1^e^)
Mortality 11,055 (6 RCTs)	Not serious	Not serious	Not serious	Serious^c^	Strong association^e^	⨁⨁⨁⨁ High	4826/5358 (90.1%)	5148/5697 (90.4%)	RR 1.00 (0.99 to 1.01)	0 fewer per 1,000 (from 9 fewer to 9 more)	Imprecision (−1^c^), Upgraded: strong association (+1^e^)
Survival rate 11,055 (6 RCTs)	Not serious	Not serious	Not serious	Serious^c^	Strong association^e^	⨁⨁⨁⨁ High	532/5358 (9.9%)	549/5697 (9.6%)	RR 0.98 (0.88 to 1.09)	2 fewer per 1,000 (from 12 fewer to 9 more)	Imprecision (−1^c^), Upgraded: strong association (+1^e^)
Airway establishment time 944 (14 RCTs)	Very serious^a^	Very serious^b^	Not serious	Not serious	None^f^	⨁◯◯◯ Very Low	474	470		MD 86.95 SD lower (103.00 lower to70.90 lower)	Risk of bias (−2^a^), Inconsistency (−1^b^)
Incidence rate 353 (6 RCTs)	Very serious^a^	Not serious	Not serious	Serious^c^	Strong association^e^	⨁⨁◯◯ Low	48/183 (26.2%)	10/170 (5.9%)	RR 0.25 (0.14 to 0.47)	197 fewer per 1,000 (from 226 fewer to 139 fewer)	Risk of bias (−2^a^), Imprecision (−1^c^), Upgraded: strong association (+1^e^)

**Figure 2:**
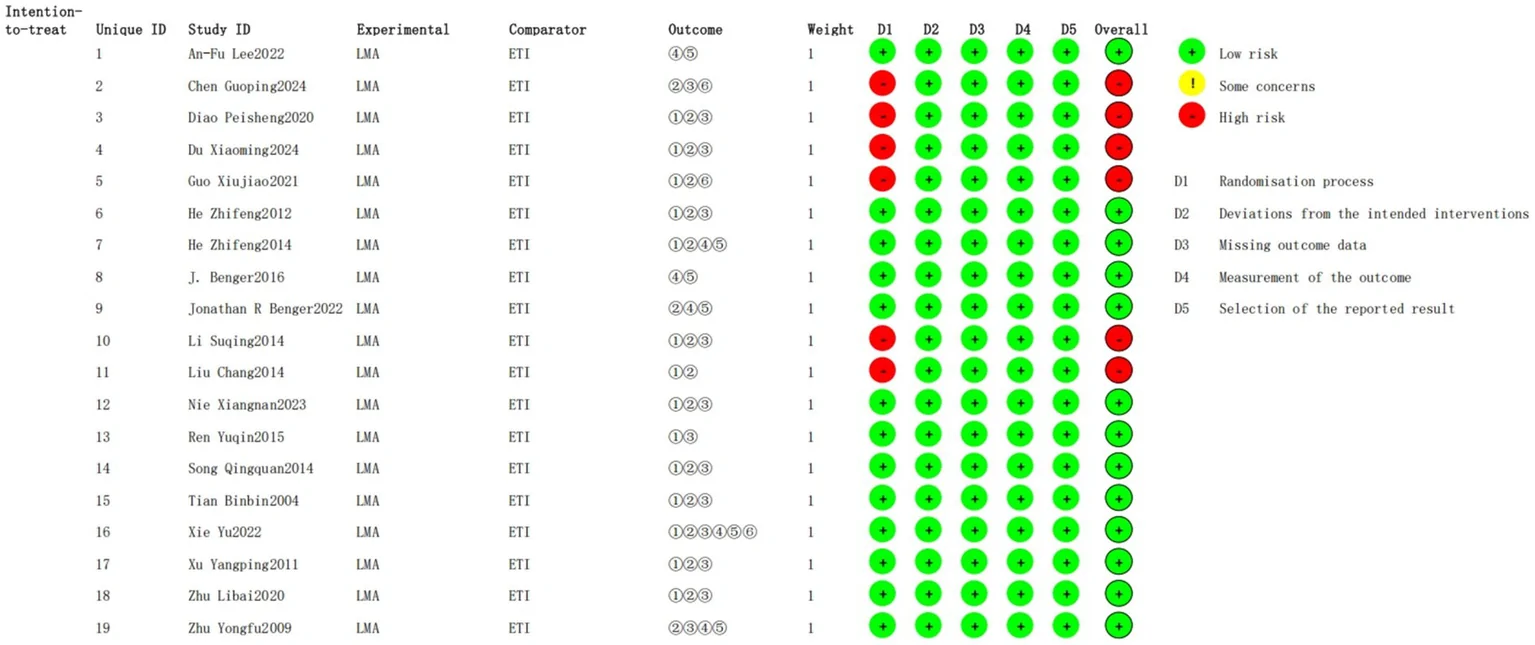
Risk of bias results for the included studies (D1: Randomization process, D2: Deviations from the intended interventions, D3: Missing outcome data, D4: Measurement of the outcome, D5: Selection of the reported result).

RR: risk ratio; SMD: standardized mean difference.

a A high risk of bias exists, as shown in Figure 2.

b obvious heterogeneity exists, as shown in Figure 4.

c A small number of articles exist, as shown in Table 2.

d Very strong association (large effect size, upgraded by 2 levels).

e Strong association (upgraded by 1 level).

f Continuous outcome; absolute effect expressed as mean difference (seconds).

## 3 Results

### 3.1 Results of study screening

A total of 1,677 relevant articles were initially identified. After removing 856 duplicates, 753 records were excluded based on title and abstract screening. Following full-text assessment, 49 additional studies were excluded as irrelevant. As illustrated in Figure 1, 19 randomized controlled trials involving 11,982 OHCA patients were ultimately included in the systematic review and meta-analysis (15, 16, 24–40).

### 3.2 Characteristics of the included randomized controlled trials

This meta-analysis included 19 studies comprising a total of 11,982 OHCA patients, with 5,823 assigned to the control group and 6,159 to the experimental group. Fifteen of these studies were published between 2014 and 2024. The sample sizes of the included studies varied considerably: most enrolled between 24 and 100 participants, five studies had sample sizes ranging from 120 to 936 (15, 25, 32, 33, 38), and one study included over 9,000 participants (16). Regarding mean age, three studies did not report this information (16, 24, 34). The overall age range across studies was approximately 27 to 112 years, though the majority of studies reported mean ages between 40 and 65 years, reflecting the typical demographic profile at increased risk for cardiac arrest. All included studies fulfilled the diagnostic criteria for prehospital cardiac arrest. Regarding adverse events, the majority of studies did not report such occurrences. Among those that did, six studies documented adverse events: aspiration of gastric contents was reported in three studies (16, 24, 31), while vocal cord injury, tooth loss, and mucosal damage were described in five studies (34, 36, 37, 39, 40). One study reported respiratory obstruction and laryngeal edema (31). The basic characteristics of the included studies are summarized in Table 2. To facilitate a concise overview of the evidence, the key meta-analysis findings across all outcomes are summarized in Table 3, including the number of studies, participants, pooled effect estimates, heterogeneity, model used, and GRADE certainty of evidence.

**Table 2:** Characteristics of included RCTs investigating the effect of laryngeal mask airway (LMA) on out-of-hospital cardiac arrest.

Included study (author/year/language)	Sample size (E/C)	Average age (E/C)	Interventions		LMA diagnostic criteria	Adverse events	Outcome
			Experiment group	Control group			
An-Fu Lee, 2022 (15)	419/517	74.7 ± 38.1/72.1 ± 16.4	LMA	ETI	conform	NR	④⑤
Chen Guoping, 2024 (39)	30/30	50.23 ± 1.52/50.28 ± 1.53	LMA	ETI	conform	Vocal cord damage, tooth loss, mucosal damage, others	②③⑥
Diao Peisheng, 2020 (35)	33/33	45.12 ± 6.37/45.19 ± 6.87	LMA	ETI	conform	NR	①②③
Du Xiaoming, 2024 (40)	30/30	36.47 ± 8.57/35.20 ± 8.66	LMA	ETI	conform	Vocal cord damage, tooth loss, mucosal damage	①②③
Guo Xiujiao, 2021 (36)	38/38	40.38 ± 11.06/41.06 ± 10.38	LMA	ETI	conform	Vocal cord damage, tooth loss, mucosal damage	①②⑥
He Zhifeng, 2012 (27)	27/26	65/63	LMA	ETI	conform	NR	①②③
He Zhifeng, 2014 (31)	30/30	47.5 ± 1.28	LMA	ETI	conform	Nausea and vomiting, respiratory obstruction, aspiration of gastric contents, laryngeal edema, laryngotracheal spasm	①②④⑤
J. Benger, 2016 (33)	174/209	71/71	LMA	ETI	conform	NR	④⑤
Jonathan R Benger, 2022 (16)	4886/4410	NR	LMA	ETI	conform	Reflux, aspiration of gastric contents	②④⑤
Li Suqing, 2014 (28)	20/20	62/63	LMA	ETI	conform	NR	①②③
Liu Chang, 2014 (29)	27/19	55.6 ± 7.8/57.4 ± 8.6	LMA	ETI	conform	NR	①②
Nie Xiangnan, 2023 (38)	72/72	53.48 ± 3.51/52.63 ± 3.46	LMA	ETI	conform	NR	①②③
Ren Yuqin, 2015 (32)	64/56	65.7 ± 12.3/67.1 ± 15.1	LMA	ETI	conform	NR	①③
Song Qingquan, 2014 (30)	30/30	36.7 ± 4.3/35.9 ± 3.5	LMA	ETI	conform	NR	①②③
Tian Binbin, 2004 (24)	10/23	NR	LMA	ETI	conform	Reflux, aspiration of gastric contents	①②③
Xie Yu, 2022 (37)	12/12	51.01 ± 2.15/50.86 ± 2.18	LMA	ETI	conform	Vocal cord damage, tooth loss, mucosal damage, others	①②③④⑤⑥
Xu Yangping, 2011 (26)	27/35	57.6 ± 7.8/59.4 ± 8.6	LMA	ETI	conform	NR	①②③
Zhu Libai, 2020 (34)	50/50	NR	LMA	ETI	conform	Vocal cord damage, tooth loss, mucosal damage	①②③
Zhu Yongfu, 2009 (25)	180/183	52/57	LMA	ETI	conform	NR	②③④⑤

E/C, Experimental group/ Control group; LMA, Laryngeal mask airway; ETI, Endotracheal intubation; R, Report; NR, Not report; ① Airway establishment time; ② success rate of one-time intubation; ③ success rate of cardiopulmonary resuscitation; ④ mortality; ⑤ survival rate; ⑥ LVEF: left ventricular ejection fraction.

**Table 3:** Summary of meta-analysis results.

Outcome	No. of studies (participants)	Effect estimate (95% CI)	I^2^	Model	GRADE certainty
Primary outcomes					
CPR success rate	13 (1185)	RR 1.44 (1.25, 1.67)	40%	FEM	Moderate ⨁⨁⨁◯
Airway establishment time	14 (944)	MD − 86.95 s (−103.00, −70.90)	99%	REM	Very low ⨁◯◯◯
Secondary outcomes					
First-attempt intubation success rate	16 (7964)	RR 1.44 (1.32, 1.58)	71%	REM	High ⨁⨁⨁⨁
Mortality	6 (11,055)	RR 1.00 (0.99, 1.01)	0%	FEM	High ⨁⨁⨁⨁
Survival rate	6 (11,055)	RR 0.98 (0.88, 1.09)	23%	FEM	High ⨁⨁⨁⨁
LVEF	3 (160)	SMD 1.57 (0.65, 2.48)	82%	REM	Not assessed
Total adverse events	6 (353)	RR 0.25 (0.14, 0.47)	0%	FEM	Low ⨁⨁◯◯

RR, risk ratio; MD, mean difference; SMD, standardized mean difference; FEM, fixed-effect model; REM, random-effects model.

Total adverse events refers to the pooled estimate across all adverse event types; detailed analyses for specific subtypes (vocal cord damage, tooth loss, mucosal damage, aspiration) are reported in Section 3.6. Model selection followed the pre-specified rule described in Section 2.5.5.

### 3.3 Risk of bias (ROB) evaluation

Thirteen studies that described adequate allocation concealment and blinding procedures were rated as “low risk” of bias in the randomization domain (15, 16, 24–27, 30–34, 37, 38). Six studies did not specify the method of randomization or provide sufficient detail, leading to an assessment of “some concerns” in the randomization process (28, 29, 35, 36, 39, 40). All studies were judged as “low risk” in the following domains: deviations from intended interventions, missing outcome data, measurement of outcomes, and selection of reported results, due to appropriate outcome assessment, complete data reporting, adherence to intervention protocols, and absence of selective outcome reporting. The overall ROB assessments are presented in Figure 2.

### 3.4 Primary outcomes

#### 3.4.1 CPR success rate

**Figure 3:**
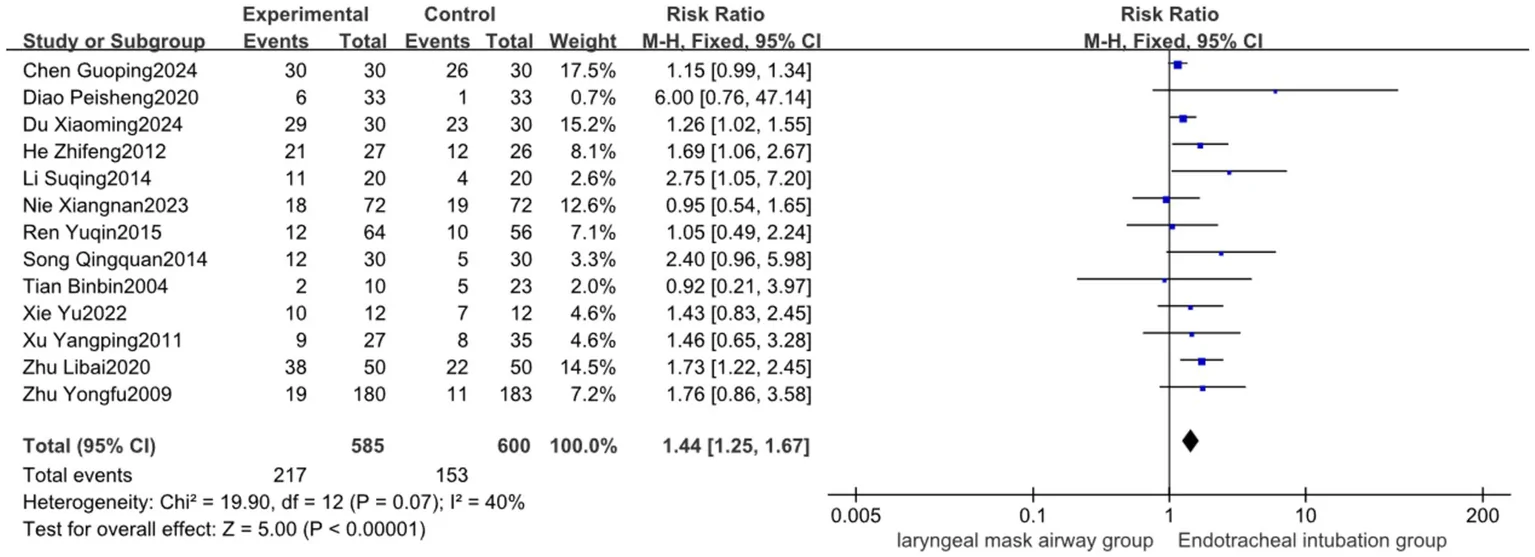
Forest plot of the CPR success rate.

Thirteen studies involving 1,185 patients reported CPR success rates (24–28, 30, 32, 34, 35, 37–40). CPR success was defined as return of spontaneous circulation (ROSC) at any time during resuscitation, consistent with our pre-specified outcome definition. In accordance with our pre-specified model selection rule (I^2^ < 50% and non-significant Q test), a fixed-effect model (FEM) was used for this analysis, as the low statistical heterogeneity (I^2^ = 40%) supported the assumption of a common treatment effect across studies. The results of the meta-analysis revealed that LMA presented a higher CPR success rate than did ETI (RR = 1.44; 95% CI (1.25, 1.67); *p* < 0.00001, Figure 3).

#### 3.4.2 Time to airway establishment

**Figure 4:**
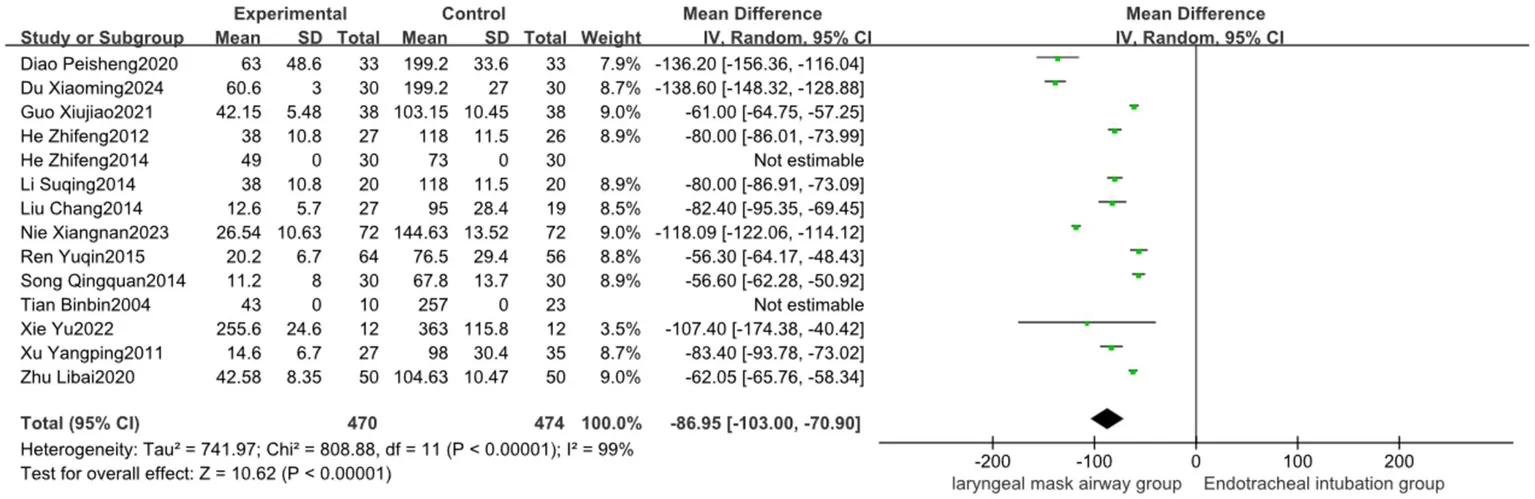
Forest plot of the time to airway establishment.

Fourteen studies involving 944 patients reported the time to airway establishment (24, 26–32, 34–38, 40). In accordance with our pre-specified analytic approach, a random-effect model (REM) was employed to account for the substantial clinical and methodological diversity across studies, which was reflected in the high statistical heterogeneity (I^2^ = 99%). The results of the meta-analysis revealed that the airway establishment time was shorter in the LMA group than in the ETI group (MD = −86.95 s; 95% CI (−103.00, −70.90); *p* < 0.00001; Figure 4). Sensitivity analyses confirmed the robustness of this finding, with no single study exerting a disproportionate influence on the pooled estimate.

### 3.5 Secondary outcomes

#### 3.5.1 First-attempt intubation success rate

**Figure 5:**
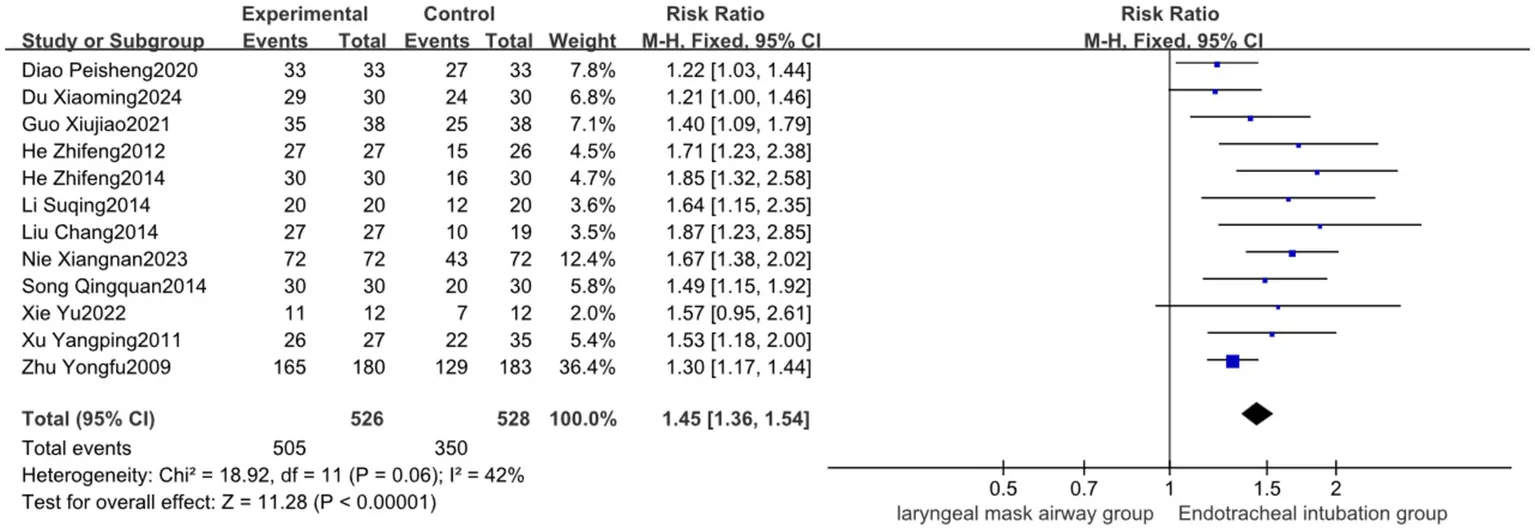
Forest plot of the first-attempt intubation success rate.

Sixteen studies involving 7,964 patients reported first-attempt intubation success rates (16, 24–31, 34–40). Consistent with our pre-specified methodology, a REM was used for the primary analysis given the moderate-to-high heterogeneity (I^2^ = 71%, Supplementary Figure 1). The results of the meta-analysis revealed that compared with ETI, LMA increased the first-attempt intubation success rate (RR = 1.44; 95% CI (1.32, 1.58); *p* < 0.00001; Supplementary Figure 1). In this primary analysis, the AIRWAYS-2 trial (16) contributed 12.2% of the statistical weight, not disproportionately. Nevertheless, to fully address concerns about the potential influence of this large trial, we performed a pre-specified sensitivity analysis excluding AIRWAYS-2. After its removal, the random-effects pooled estimate remained consistent and statistically significant (RR = 1.50; 95% CI [1.34, 1.67]; *p* < 0.00001; I^2^ = 67%; Supplementary Figure 2), confirming that the overall effect was not driven by any single study. To assess the robustness of these findings, additional sensitivity analyses were performed by sequentially excluding individual studies from the AIRWAYS-2-removed dataset. When three studies (24, 34, 39) were removed—including one study with an ETI group intubation success rate of less than 50% (34), one study with the highest success rate of primary intubation in the ETI group (39) and one study with the oldest publication date (24), —the heterogeneity decreased to 42%, permitting a fixed-effect model in accordance with our pre-specified model selection rule, while the pooled estimate remained consistent (RR = 1.45; 95% CI [1.36, 1.54]; p < 0.00001; Figure 5).

#### 3.5.2 Mortality and survival rate

**Figure 6:**
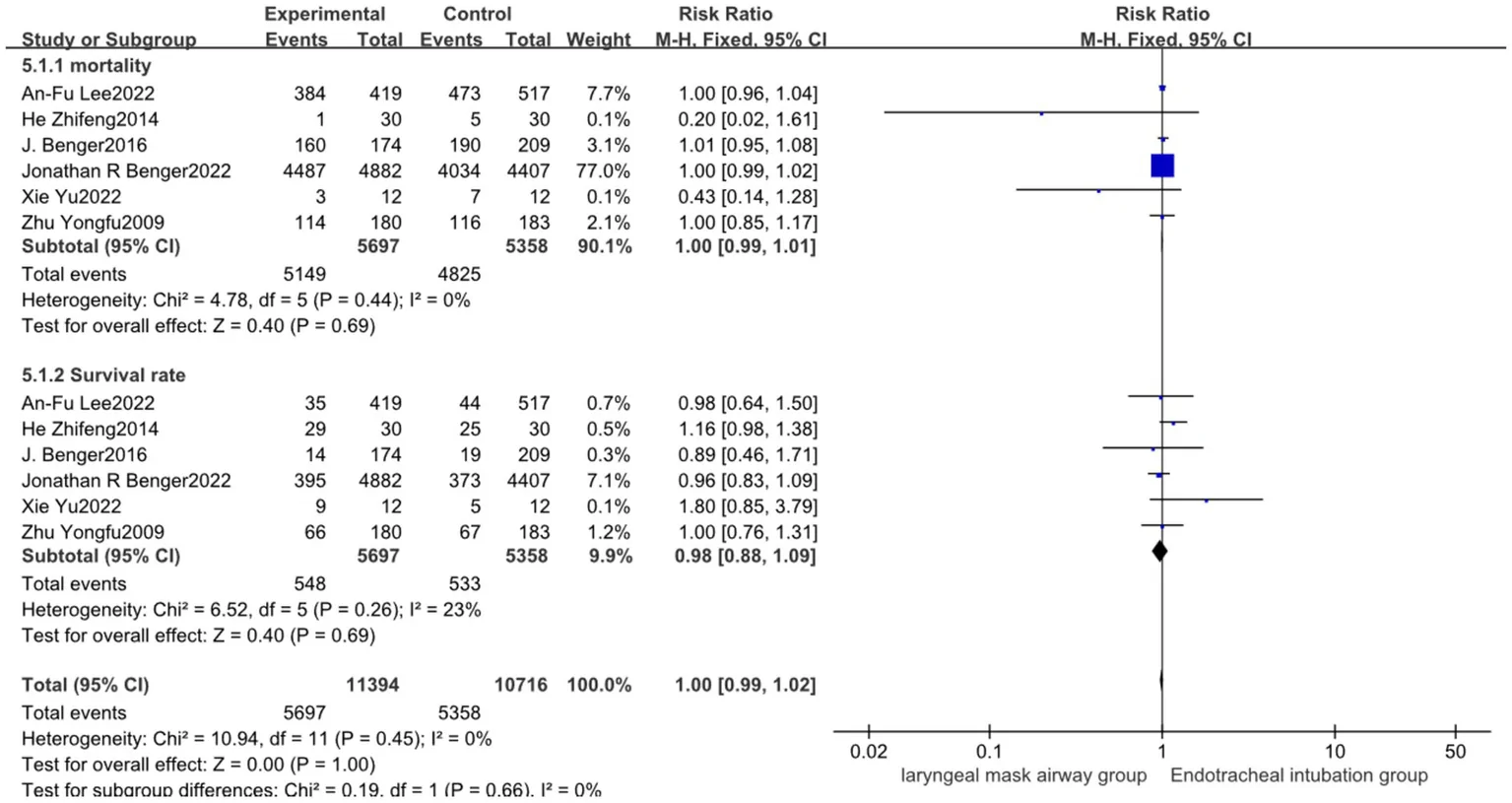
Forest plot of mortality and survival rates.

Six studies involving 11,055 patients reported mortality and survival rates (15, 16, 25, 31, 33, 37). In accordance with our pre-specified definitions, mortality was all-cause death before hospital discharge (with 30-day mortality accepted as a proxy when discharge data were unavailable), and survival rate was survival to hospital discharge. Following our pre-specified approach, a FEM was applied, as the low heterogeneity (mortality I^2^ = 0%; survival rate I^2^ = 23%) suggested that the treatment effects were consistent across studies. The results of the meta-analysis suggested that LMA was similar to ETI in improving the survival rate (RR = 0.98; 95% CI (0.88, 1.09); *p* = 0.69; Figure 6), and reducing mortality (RR = 1.00; 95% CI (0.99, 1.01); *p* = 0.69; Figure 6). In this analysis, the AIRWAYS-2 trial (16) contributed the majority of the statistical weight. To transparently demonstrate whether the pooled estimates were driven by this single large trial, we performed a pre-specified sensitivity analysis excluding AIRWAYS-2. After its removal, the fixed-effect estimates remained essentially unchanged (mortality: RR = 0.99; 95% CI [0.96, 1.03]; *p* = 0.70; survival: RR = 1.04; 95% CI [0.87, 1.24]; *p* = 0.70; Supplementary Figure 3), with low residual heterogeneity (mortality I^2^ = 22%; survival I^2^ = 3%) and no single study dominating the weights.

#### 3.5.3 LVEF

**Figure 7:**
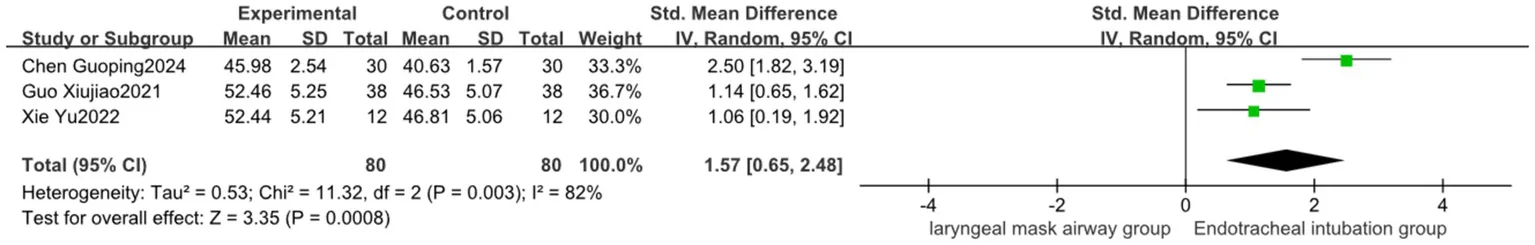
Forest plot of LVEF.

Three studies involved 160 patients reporting LVEF (36, 37, 39). In line with our pre-specified strategy, a REM was used for the primary analysis to account for the high statistical heterogeneity (I^2^ = 82%). The results of the meta-analysis suggested that the LMA group is superior to the ETI group in improving LVEF (SMD = 1.57; 95% CI (0.65, 2.48); *p* = 0.0008; Figure 7) Sensitivity analyses confirmed the robustness of this finding, with no single study exerting a disproportionate influence on the pooled estimate.

### 3.6 Comparison of the safety of adverse events

**Figure 8:**
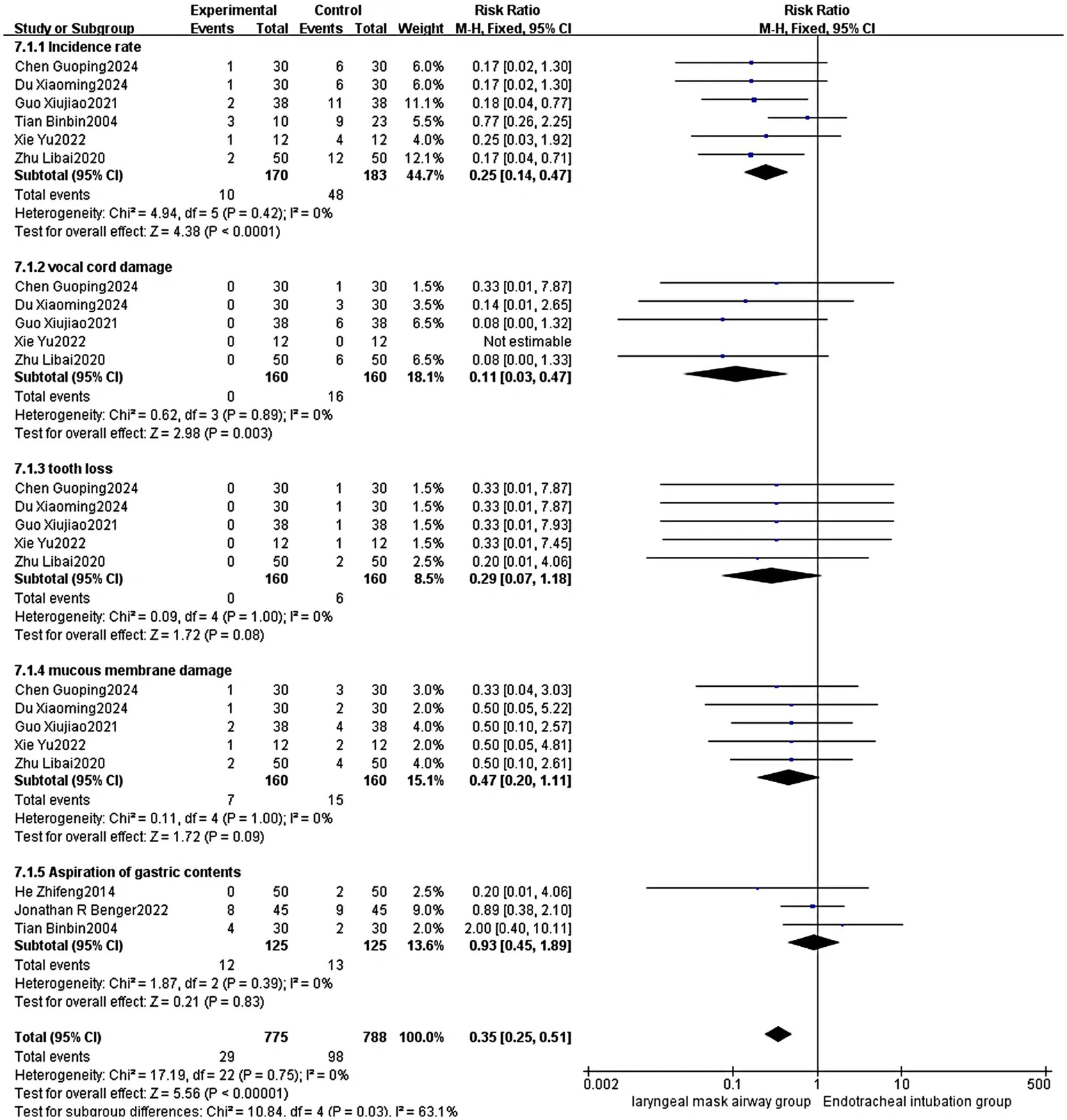
Forest plot of adverse events.

Adverse events were reported in six studies involving 353 patients (24, 34, 36, 37, 39, 40) (Table 2). As pre-specified in our subgroup analysis (Section 2.5.5), we calculated pooled estimates separately for each adverse event subtype. According to our pre-specified protocol, a FEM was used for these analyses, as the little I^2^ values (ranging from 0 to 0%) indicated negligible statistical heterogeneity across studies for each adverse event type. The results of the meta-analysis revealed that the LMA group had significantly lower risks than the ETI group in terms of total adverse events (RR = 0.25; 95% CI (0.14, 0.47); *p* < 0.0001) and vocal cord damage (RR = 0.11; 95% CI (0.03, 0.47); *p* = 0.003). Although numerical reductions in tooth loss (RR = 0.29; 95% CI (0.07, 1.18); *p* = 0.08) and mucous membrane damage (RR = 0.47; 95% CI (0.20, 1.11); *p* = 0.09) were detected, these differences did not reach statistical significance. Importantly, there was no statistically significant difference between the groups in the incidence of aspiration of gastric contents (RR = 0.93; 95% CI (0.45, 1.89); *p* = 0.83) (Figure 8). These results suggest that LMA may offer a more favorable profile with respect to mechanical airway injury compared to ETI. However, these safety findings must be interpreted with considerable caution. The total number of events was small for several specific complications (e.g., only 6 events of tooth loss and 22 events of mucosal damage across all studies), and all safety analyses included only six studies with a combined total of 353 patients. The resulting wide confidence intervals and marginal *p*-values indicate that the estimates are fragile, and the addition of even a small number of new events in future studies could substantially alter the pooled estimates. Consequently, while the direction of effect consistently favors LMA, these data do not support an unqualified conclusion that LMA is universally safer than ETI across all adverse event categories.

### 3.7 Findings from the publishing bias evaluation

**Figure 9:**
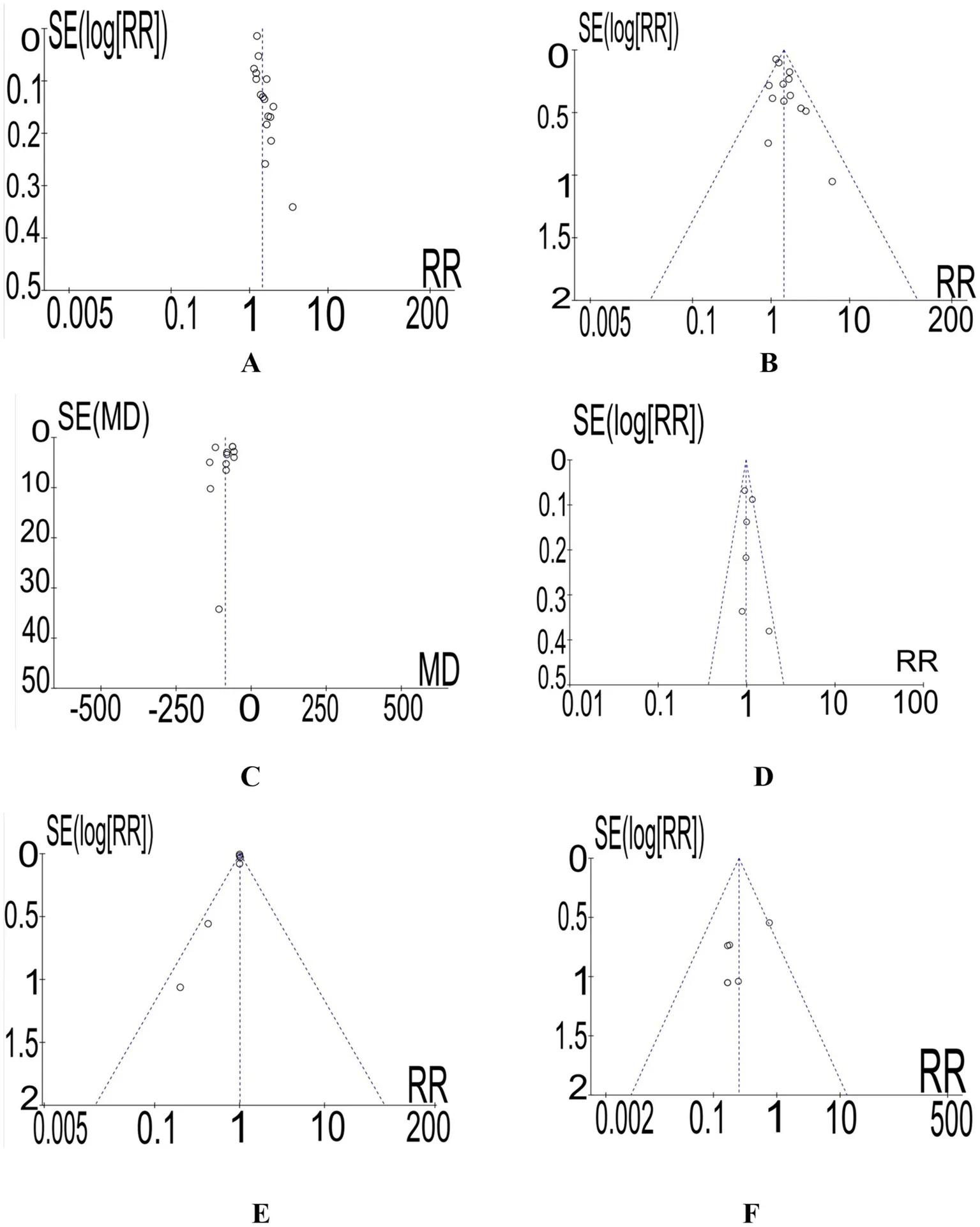
Funnel plot for publication bias assessment. **(A)** First-attempt intubation success rate publication bias assessment, **(B)** success rate of CPR publication bias assessment, **(C)** airway establishment time publication bias assessment, **(D)** survival rate publication bias assessment, **(E)** mortality publication bias assessment, and **(F)** incidence rate publication bias assessment.

We evaluated the impact of publication bias on the following metrics: first-attempt intubation success rate, CPR success rate, airway establishment time, survival rate, mortality, and incidence rate. For outcomes with ten or more included studies (first-attempt intubation success rate, CPR success rate, and airway establishment time), funnel plots were visually inspected for potential asymmetry, and the generally symmetric distribution of data points suggested no obvious evidence of publication bias. For survival rate, mortality, and incidence rate, each of which included fewer than ten studies, the funnel plots are presented solely as a descriptive visual supplement; no formal interpretation of asymmetry is warranted given the limited number of studies, as the statistical power to distinguish true asymmetry from chance is low. Consequently, even descriptive visual inspection of these funnel plots should be interpreted with caution, as the limited number of studies reduces the reliability of any asymmetry assessment. In all cases, the data points were largely centralized and did not fall outside the funnel boundaries. However, due to the lack of access to unpublished study protocols or trial registrations, the possibility of reporting bias cannot be fully excluded (Figure 9). Each sub-panel of Figure 9 is also provided as an individual high-resolution supplementary figure to facilitate clearer inspection: Supplementary Figure 4 (first-attempt intubation success rate), Supplementary Figure 5 (CPR success rate), Supplementary Figure 6 (airway establishment time), Supplementary Figure 7 (survival rate), Supplementary Figure 8 (mortality), and Supplementary Figure 9 (incidence rate).

### 3.8 Outcomes of trial sequential analysis (TSA)

**Figure 10:**
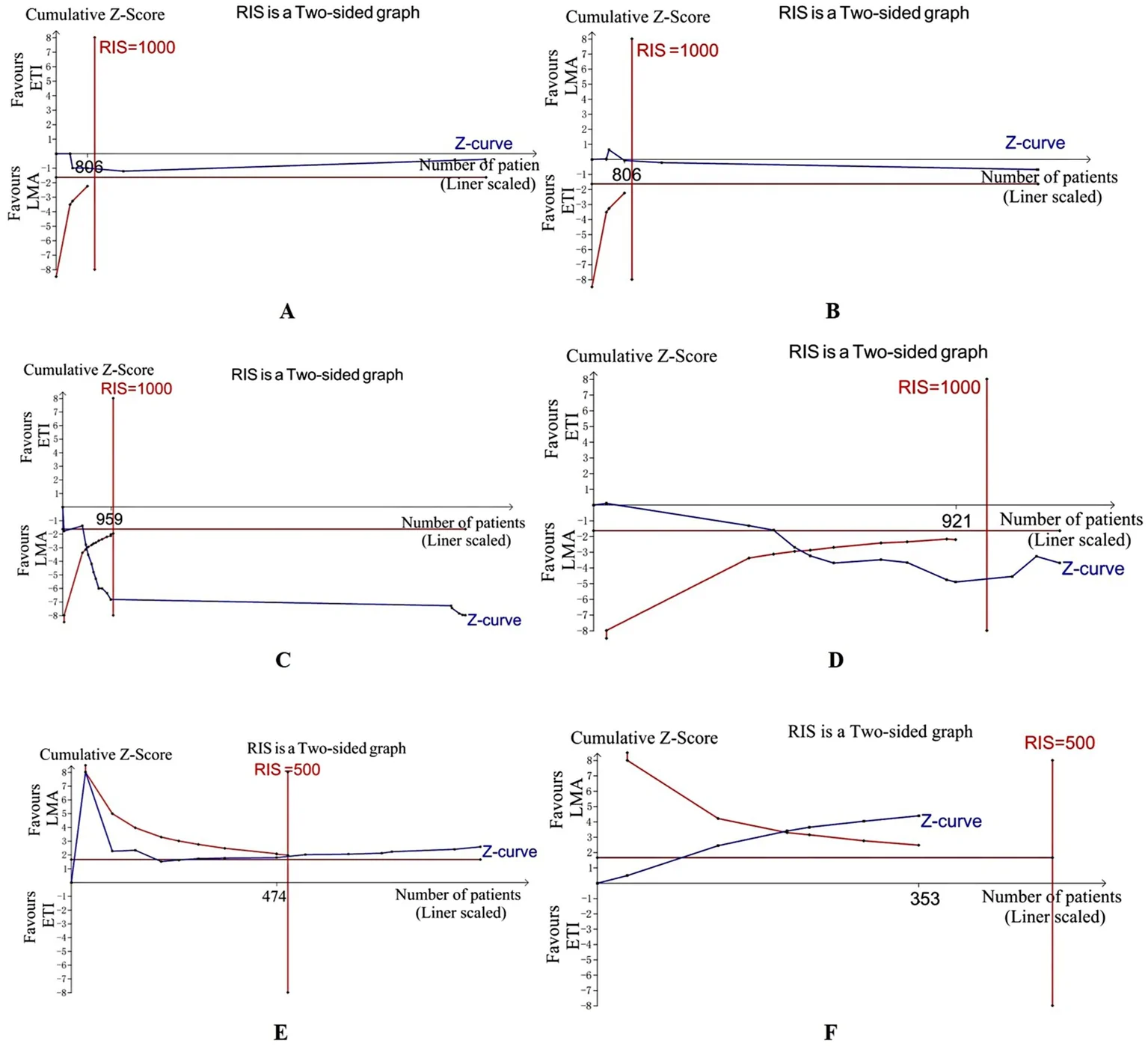
TSA results. **(A)** Survival rate, **(B)** Mortality, **(C)** One-time intubation success rate, **(D)** Success rate of cardiopulmonary resuscitation, **(E)** Airway establishment time, **(F)** Incidence rate.

For survival and mortality outcomes, although the cumulative Z-curve reached the RIS line, it did not cross the TSA threshold, indicating that while LMA may be associated with improved survival and reduced mortality compared to ETI in OHCA, the current evidence remains suggestive and requires confirmation in additional trials. For morbidity, the cumulative Z-curve crossed the TSA threshold but did not exceed the RIS, suggesting that the potential superiority of LMA in reducing morbidity should be interpreted with caution due to insufficient cumulative evidence. In contrast, for first-attempt success rate, CPR success rate, and time to airway establishment, the cumulative Z-curve crossed both the TSA monitoring boundary and the RIS line, and the cumulative information reached the expected values, demonstrating robust evidence in favor of LMA over ETI without the need for further studies (Figure 10). The individual TSA plots corresponding to each sub-panel of Figure 10 are also provided as high-resolution supplementary figures: Supplementary Figure 10 (survival rate), Supplementary Figure 11 (mortality), Supplementary Figure 12 (first-attempt intubation success rate), Supplementary Figure 13 (CPR success rate), Supplementary Figure 14 (airway establishment time), and Supplementary Figure 15 (incidence rate).

### 3.9 Quality of the evidence

The certainty of meta-outcome evidence was evaluated via the Grading of Recommendations, Assessments, Developments, and Evaluations (GRADE) approach on the basis of the risks of bias, inconsistencies, imprecision and publication bias. The results revealed that the quality of evidence ranged from “very low” to “high.” The evidence quality for the one-time intubation success rate was rated as high due to the very serious risk of bias (ROB) which was fully offset by a very strong association (upgrade +2). With consideration of serious imprecision and a strong association (upgrade +1), the evidence quality for mortality and survival rates was assessed as high. The success rate of cardiopulmonary resuscitation received a moderate evidence quality rating based on very serious ROB partially offset by a strong association. Incidence rate was classified as low evidence quality owing to very serious ROB, serious imprecision, and a strong association. The time to airway establishment was rated as very low evidence quality due to very serious ROB and very serious inconsistency; no upgrading was applied. Downgrading was primarily attributed to elevated ROB (Figure 2), substantial heterogeneity among the included studies (I^2^ > 75%; Figure 4), and the limited number of incorporated studies (Table 2). The specific downgrade and upgrade steps for each outcome are documented transparently in the Summary of Findings table (Table 1).

## 4 Discussion

### 4.1 Key findings: clinical advantages of LMA

This systematic review and meta-analysis provided a comprehensive comparison of LMA versus ETI for airway management in patients with OHCA, offering high-quality evidence regarding the clinical advantages of LMA in prehospital resuscitation. Unlike prior meta-analyses that combined randomized and non-randomized studies and reported conflicting conclusions, the present work exclusively pools RCTs, incorporates recent trials, and includes pre-specified sensitivity analyses (including the exclusion of the largest trial for relevant outcomes) and TSA to ensure robustness. Based on 19 RCTs involving 11,982 patients, this study represents one of the largest and methodologically most rigorous meta-analyses to date comparing LMA and ETI in the OHCA setting, demonstrating notable improvements in both analytical robustness and clinical relevance over prior research. It is important to distinguish between procedural efficiency outcomes (airway establishment time, first-attempt success, CPR success) and patient-centered survival outcomes (survival to discharge, mortality). Our analysis shows a consistent pattern: LMA improves procedural metrics, but these advantages do not translate into a long-term survival benefit. The results indicate that the use of LMA significantly reduced the time required to establish an airway (MD: −86.95 s; 95% CI: −103.00, −70.90; *p* < 0.00001). This efficiency contributes to minimized interruptions in chest compressions and better maintenance of cerebral perfusion—a mechanistic insight further supported by improved LVEF (SMD: 1.57; 95% CI: 0.65, 2.48; *p* < 0.0001). Moreover, LMA was associated with a higher first-attempt success rate (RR: 1.44; 95% CI: 1.32, 1.58; *p* < 0.00001), reinforcing its practical utility and technical superiority in prehospital emergency care.

These procedural advantages were associated with significant short-term clinical benefits, as the LMA group exhibited a 44% higher CPR success rate (defined as ROSC at any time during resuscitation) compared to the ETI group (RR: 1.44; 95% CI: 1.25, 1.67; *p* < 0.00001), a finding further supported by trial sequential analysis in which the cumulative Z-curve crossed the required information size (RIS) boundary. From a pathophysiological perspective, the shorter airway establishment time achieved with LMA directly reduces the duration of chest compression interruptions, thereby preserving coronary and cerebral perfusion pressure during the critical early phase of resuscitation (41). This hemodynamic benefit is reflected in the significantly improved post-resuscitation LVEF observed in the LMA group, providing a plausible mechanistic link between procedural efficiency and short-term cardiac function. Nevertheless, these short-term gains did not yield significant improvements in long-term outcomes: both survival (RR: 0.98; 95% CI: 0.88, 1.09; *p* = 0.69) and mortality (RR: 1.00; 95% CI: 0.99, 1.01; *p* = 0.69) were comparable between the two groups. Thus, while LMA offers clear operational advantages, these do not translate into a survival benefit, and the device should be viewed as an effective alternative rather than a replacement for ETI in all circumstances.

### 4.2 Safety outcomes and persistent uncertainties

The safety profiles of ETI and LMA in prehospital emergency care remain a subject of ongoing debate, with considerable inconsistency in reported outcomes across studies. Previous research has highlighted the potential superiority of ETI in airway protection, largely attributable to its subglottic sealing mechanism, which reduces the risk of aspiration during positive-pressure ventilation—especially in high-risk clinical contexts such as traumatic brain injury or patients with an elevated risk of vomiting (13, 42). Conversely, LMA has been promoted for its rapid insertion and reduced operator skill requirement. However, traditional concerns have included its limited sealing pressure (typically 18–22 cmH₂O) and potential for gastric insufflation in non-fasted patients (43). In contrast, our findings indicate that the incidence of gastric content reflux with LMA was marginally lower than with ETI (RR = 0.93; *p* = 0.83), challenging conventional assumptions regarding aspiration risk (44). This improved safety profile is likely due to enhancements in second-generation LMA devices, which incorporate features such as dedicated esophageal drainage channels to reduce the risk of regurgitation (45). Furthermore, the LMA group demonstrated a significantly lower overall incidence of adverse events (RR = 0.25; *p* < 0.0001), including an 89% reduction in vocal cord injuries (RR = 0.11; *p* = 0.003). These findings are consistent with previous studies emphasizing the reduced mechanical trauma associated with LMA use on airway structures (43). The present results contrast with earlier meta-analyses that reported superior survival rates with ETI in the context of out-of-hospital cardiac arrest (13), highlighting that the choice of airway device should consider both clinical factors—such as aspiration risk and operator proficiency—and ongoing technological advancements. Our study reinforces the safety profile of modern LMA devices with respect to airway trauma and helps reconcile earlier conflicting evidence through improved device design. Nevertheless, given the extremely sparse event counts for several adverse outcomes—particularly vocal cord damage, tooth loss, and mucosal damage, where zero or few events were reported in the LMA arm—the precision of these safety estimates is limited. These findings should be viewed as hypothesis-generating rather than definitive, and future trials with systematic adverse event reporting are needed to confirm the safety advantages suggested by the current data.

### 4.3 Efficacy–survival paradox and system-level heterogeneity

Notably, the observed discrepancy between improved CPR success and unchanged survival was not attributable to differences in outcome definitions across studies, as standardized definitions were applied throughout the meta-analysis. This dichotomy reveals a critical consideration: while the “golden hour” interventions reflected in our metrics—such as airway establishment time and CPR success rate—are necessary, they may be insufficient to ensure ultimate survival, which is also strongly influenced by the quality of post-resuscitation care and preexisting comorbidities (46, 47).

Three main limitations should be acknowledged. First, the considerable heterogeneity observed in airway timing metrics (I^2^ > 75%) likely stems from both clinical variation (such as differences in EMS response times across regions) and methodological inconsistencies in time recording—a significant confounder that underscores the need for standardized measurement in future studies. Second, notable geographic imbalance must be considered: although Western populations accounted for 80.1% of all participants (9,679/11,982), these individuals were drawn from only two RCTs (10.5% of included studies) (16, 33), most notably the AIRWAYS-2 trial (16), which contributed 9,296 cases. In contrast, 17 Asian trials (89.5% of studies) enrolled 2,303 participants (19.9% of the total), illustrating a pronounced inverse relationship between the number of studies and participant distribution across regions. This skewed distribution raises concerns regarding the generalizability of the findings: (1) Western data largely originate from paramedic-led emergency systems with standardized protocols, while Asian trials reflect varied, physician-driven EMS models; (2) large-scale trials such as AIRWAYS-2 may disproportionately influence pooled outcomes, reflecting specific settings where ETI success rates were notably high (72% compared to 33–61% in typical Western systems) (48, 49). Substantial differences in operator experience—with Western paramedics averaging 50 intubations per year versus over 200 among Asian physicians (50–52)—further limit the extrapolation of airway management efficacy across these divergent healthcare contexts.

### 4.4 Methodological innovations and limitations

Nevertheless, several limitations persist, including clinical heterogeneity arising from variations in intervention protocols (e.g., differences in LMA generations) and disparities in operator expertise (paramedic- versus physician-performed intubation). To assess the potential disproportionate influence of the largest trial, we performed pre-specified sensitivity analyses excluding AIRWAYS-2 from all outcomes to which it contributed; the pooled estimates remained consistent, confirming that our findings were not driven by this single study. Although funnel plots indicated a low risk of publication bias for primary outcomes, the potential influence of unpublished negative studies cannot be ruled out entirely. Trial sequential analysis (TSA) confirmed sufficient evidence supporting procedural outcomes; however, additional high-quality RCTs are needed to establish definitive conclusions regarding survival benefits. Furthermore, two of the 19 included studies (15, 16) were cluster-randomized trials; although their primary analyses appropriately accounted for clustering and our sensitivity analysis excluding the largest cluster-randomized trial (AIRWAYS-2) did not alter any conclusion, residual design effects cannot be entirely excluded.

## 5 Conclusion

This comprehensive systematic review and meta-analysis, which included 19 randomized controlled trials and over 11,000 OHCA patients, demonstrates that LMA holds significant operational and short-term clinical benefits over ETI in prehospital cardiac arrest management. The use of LMA was associated with a shorter time to airway establishment, a higher first-attempt success rate, and improved CPR success rates, highlighting its procedural efficiency and practicality in emergency settings. Moreover, LMA was associated with a lower incidence of airway-related adverse events, particularly vocal cord injury, although these safety findings are based on a limited number of events and require confirmation in larger studies.

Despite these operational advantages, no statistically significant differences in long-term survival were identified between the LMA and ETI groups, suggesting that factors subsequent to initial airway management—such as the quality of post-resuscitation care—critically influence ultimate patient outcomes. These results challenge the conventional preference for ETI as the gold standard and support the broader integration of LMA into clinical practice, especially in environments where proficiency in ETI is limited or where delays in securing an airway may compromise cerebral perfusion. However, this integration should be tempered by the understanding that improved procedural efficiency has not been shown to improve long-term survival, and device selection should prioritize patient-centered outcomes.

It should be noted, however, that considerable heterogeneity among the included studies, as well as geographic variability and disparities in provider experience, may limit the generalizability of these findings. Future large-scale, multicenter randomized controlled trials incorporating rigorous neurological outcome assessments and standardized post-resuscitation care protocols are urgently needed to better elucidate the influence of airway device selection on long-term survival and functional recovery. Notwithstanding these limitations, the current analysis suggests that LMA is a safe and effective alternative to ETI in out-of-hospital cardiac arrest, offering procedural advantages in airway establishment time, first-attempt success, and CPR success rate, without compromising survival or increasing aspiration risk. Given the absence of a mortality benefit, LMA should not be regarded as universally superior to ETI, but rather as an acceptable option that may be particularly valuable in settings where rapid airway control is critical or where ETI proficiency varies. Clinical protocols should balance these procedural efficiencies with the lack of demonstrated survival advantage when selecting the optimal airway strategy for individual patients. In summary, the current evidence indicates that LMA may offer procedural advantages and fewer airway-related complications compared with ETI, while evidence for a survival benefit remains insufficient.

## Data Availability

Publicly available datasets were analyzed in this study. This data can be found at: the China Science and Technology Journal Database (VIP), the Wanfang Database, CBM, CNKI, Embase, the Cochrane Library, PubMed, and the Web of Science.

## Glossary

CBM: China Biology Medicine disc
CI: confidence interval
CNKI: China National Knowledge Infrastructure
CPR: cardiopulmonary resuscitation
ETI: endotracheal intubation
FEM: Fixed effect model
GRADE: Grading of Recommendations, Assessment, Development, and Evaluation
ICU: intensive care unit
LMA: laryngeal mask airway
LVEF: left ventricular ejection fraction
OHCA: out-of-hospital cardiac arrest
PRISMA: Preferred Reporting Items for Systematic Reviews and Meta-Analyses
RCT: randomized controlled trial
RIS: required information size
REM: random effect model
ROB: risk of bias
ROSC: return of spontaneous circulation
RR: relative risk
SMD: standardized mean difference
TSA: trial sequential analysis
VIP: China Science and Technology Journal Database
