# Supplementary material for: Effect of laryngeal mask airway versus endotracheal intubation on survival in out-of-hospital cardiac arrest: a meta-analysis of randomized controlled trials
Source: Front Public Health. 2026 Aug 17;14:1877439. doi: 10.3389/fpubh.2026.1877439 (PMC13526931; doi:10.3389/fpubh.2026.1877439)
Supplement: Supplementary file 1 [file Supplementary_file_1.docx]

**Supplementary Figure 1**: Forest plot of the first-attempt intubation success rate

**Supplementary Figure 2:** Sensitivity analysis of first-attempt intubation success rate excluding the AIRWAYS-2 trial.

**Supplementary Figure 3.** Sensitivity analysis of mortality and survival rates excluding the AIRWAYS-2 trial (fixed-effect model)

**Supplementary Figure 4.** Funnel plot for publication bias assessment: first-attempt intubation success rate (corresponding to Figure 9A).

**Supplementary Figure 5.** Funnel plot for publication bias assessment: CPR success rate (corresponding to Figure 9B).

**Supplementary Figure 6.** Funnel plot for publication bias assessment: airway establishment time (corresponding to Figure 9C).

**Supplementary Figure 7.** Funnel plot for publication bias assessment: survival rate (corresponding to Figure 9D).

Note: This funnel plot is presented for descriptive visualization only; formal assessment of publication bias is not warranted due to the limited number of studies (<10).

**Supplementary Figure 8.** Funnel plot for publication bias assessment: mortality (corresponding to Figure 9E).

Note: This funnel plot is presented for descriptive visualization only; formal assessment of publication bias is not warranted due to the limited number of studies (<10).

**Supplementary Figure 9.** Funnel plot for publication bias assessment: incidence rate of adverse events (corresponding to Figure 9F).

Note: This funnel plot is presented for descriptive visualization only; formal assessment of publication bias is not warranted due to the limited number of studies (<10).

**Supplementary Figure 10.** Trial sequential analysis: survival rate (corresponding to Figure 10A).

**Supplementary Figure 11.** Trial sequential analysis: mortality (corresponding to Figure 10B).

**Supplementary Figure 12.** Trial sequential analysis: one-time intubation success rate (corresponding to Figure 10C).

**Supplementary Figure 13.** Trial sequential analysis: CPR success rate (corresponding to Figure 10D).

**Supplementary Figure 14.** Trial sequential analysis: airway establishment time (corresponding to Figure 10E).

**Supplementary Figure 15.** Trial sequential analysis: incidence rate of adverse events (corresponding to Figure 10F).
