# Supplementary material for: Effect of laryngeal mask airway versus endotracheal intubation on survival in out-of-hospital cardiac arrest: a meta-analysis of randomized controlled trials
Source: Front Public Health. 2026 Aug 17;14:1877439. doi: 10.3389/fpubh.2026.1877439 (PMC13526931; doi:10.3389/fpubh.2026.1877439)
Supplement: Supplementary file 2 [file Supplementary_file_2.docx]

**The search strategy of CNKI**

检索日期：自建库至2025年5月31日

(SU %= '急救医疗服务' OR SU %= '院外' OR SU %= '院前' OR SU %= '现场急救' OR SU %= '救护车上急救' OR SU %= '现场医疗' OR SU %= '移动医疗' OR SU %= '入院前急救' OR SU %= '急诊中心' OR SU %= '急救医护' OR SU %= '急救卫生保健服务' OR SU %= '急诊医疗服务') AND (SU %= '猝死, 心脏' OR SU %= '心脏骤停' OR SU %= '心搏骤停' OR SU %= '心跳停止' OR SU %= '心室颤动' OR SU %= '心室停顿' OR SU %= '心源性猝死' OR SU %= '心脏停搏' OR SU %= '心脏性猝死' OR SU %= '心肺按压' OR SU %= '心肺复苏' OR SU %= '心脏停顿' OR SU %= '无脉性电活动' OR SU %= '无脉搏电活动' OR SU %= '心脏猝死' OR SU %= '心搏停止' OR SU %= '心肺骤停' OR SU %= '心跳骤停' OR SU %= '心肺脑复苏' OR SU %= '心脏骤停复苏' OR SU %= '心脏病猝死' OR SU %= '心性猝死' OR SU %= '心室纤颤') AND (SU %= '喉罩通气' OR SU %= '喉罩' OR SU %= '喉罩气道' OR SU %= 'LMA' OR SU %= '喉罩通气道' OR SU %= '喉部通气罩' OR SU %= '喉部气道罩' OR SU %= '喉部通气道' OR SU %= '喉罩呼吸支持' OR SU %= '喉部通气装置' OR SU %= '人工气道喉罩' OR SU %= '喉面罩' OR SU %= '喉面罩导气管' OR SU %= '气道管理') AND (SU %= '气管插管' OR SU %= '气管通气' OR SU %= '高级气道管理' OR SU %= '气道插管' OR SU %= '经口气管插管' OR SU %= '经鼻气管插管' OR SU %= '气管内插管' OR SU %= '快速气管插管' OR SU %= '直接喉镜插管' OR SU %= '气管内导管' OR SU %= '呼吸道插管' OR SU %= '人工气道建立' OR SU %= '机械通气插管' OR SU %= '气管导管插入' OR SU %= '气管内导管置入' OR SU %= '呼吸道插管' OR SU %= '插管法, 气管内' OR SU %= '插管法气管内' OR SU %= '插管术气管内')

**The search strategy of CBM**

((("急救医护"[常用字段:智能] OR "急诊医疗服务"[常用字段:智能] OR "急救卫生保健服务"[常用字段:智能] OR "急救医疗服务"[常用字段:智能] OR "院外"[常用字段:智能] OR "院前"[常用字段:智能] OR "现场急救"[常用字段:智能] OR "救护车上急救"[常用字段:智能] OR "现场医疗"[常用字段:智能] OR "移动医疗"[常用字段:智能] OR "入院前急救"[常用字段:智能] OR "急诊中心"[常用字段:智能])) AND ((("心肺脑复苏"[常用字段:智能] OR "心脏骤停复苏"[常用字段:智能] OR "心脏病猝死"[常用字段:智能] OR "心性猝死"[常用字段:智能] OR "心室纤颤"[常用字段:智能] OR "心肺按压"[常用字段:智能] OR "心肺复苏"[常用字段:智能] OR "心脏停顿"[常用字段:智能] OR "无脉性电活动"[常用字段:智能] OR "无脉搏电活动"[常用字段:智能] OR "心脏猝死"[常用字段:智能] OR "心搏停止"[常用字段:智能] OR "心肺骤停"[常用字段:智能] OR "心跳骤停"[常用字段:智能] OR "猝死，心脏"[常用字段:智能] OR "心脏骤停"[常用字段:智能] OR "心搏骤停"[常用字段:智能] OR "心跳停止"[常用字段:智能] OR "心室颤动"[常用字段:智能] OR "心室停顿"[常用字段:智能] OR "心源性猝死"[常用字段:智能] OR "心脏停搏"[常用字段:智能] OR "心脏性猝死"[常用字段:智能]))) AND ((("喉部通气装置"[常用字段:智能] OR "人工气道喉罩"[常用字段:智能] OR "喉面罩"[常用字段:智能] OR "喉面罩导气管"[常用字段:智能] OR "气道管理"[常用字段:智能] OR "喉罩通气"[常用字段:智能] OR "喉罩"[常用字段:智能] OR "喉罩气道"[常用字段:智能] OR "LMA"[常用字段:智能] OR "喉罩通气道"[常用字段:智能] OR "喉部通气罩"[常用字段:智能] OR "喉部气道罩"[常用字段:智能] OR "喉部通气道"[常用字段:智能] OR "喉罩呼吸支持"[常用字段:智能]))) AND (("气管插管"[常用字段:智能] OR "气管通气"[常用字段:智能] OR "高级气道管理"[常用字段:智能] OR "气道插管"[常用字段:智能] OR "经口气管插管"[常用字段:智能] OR "经鼻气管插管"[常用字段:智能] OR "气管内插管"[常用字段:智能] OR "快速气管插管"[常用字段:智能] OR "直接喉镜插管"[常用字段:智能] OR "气管内导管"[常用字段:智能] OR "呼吸道插管"[常用字段:智能] OR "人工气道建立"[常用字段:智能] OR "机械通气插管"[常用字段:智能] OR "气管导管插入"[常用字段:智能] OR "气管内导管置入"[常用字段:智能] OR "插管法, 气管内"[常用字段:智能] OR "插管法气管内"[常用字段:智能] OR "插管术气管内"[常用字段:智能])) AND ("日期: 建库-20250531"[常用字段:智能])

**The search strategy of VIP**

((ME=急救医疗服务 OR ME=院外 OR ME=院前 OR ME=现场急救 OR ME=救护车上急救 OR ME=现场医疗 OR ME=移动医疗 OR ME=入院前急救 OR ME=急诊中心 OR ME=急救医护 OR ME=急救卫生保健服务 OR ME=急诊医疗服务)) AND ((ME=猝死, 心脏 OR ME=心脏骤停 OR ME=心搏骤停 OR ME=心跳停止 OR ME=心室颤动 OR ME=心室停顿 OR ME=心源性猝死 OR ME=心脏停搏 OR ME=心脏性猝死 OR ME=心肺按压 OR ME=心肺复苏 OR ME=心脏停顿 OR ME=无脉性电活动 OR ME=无脉搏电活动 OR ME=心脏猝死 OR ME=心搏停止 OR ME=心肺骤停 OR ME=心跳骤停 OR ME=心肺脑复苏 OR ME=心脏骤停复苏 OR ME=心脏病猝死 OR ME=心性猝死 OR ME=心室纤颤)) AND ((ME=喉罩通气 OR ME=喉罩 OR ME=喉罩气道 OR ME= LMA OR ME=喉罩通气道 OR ME=喉部通气罩 OR ME=喉部气道罩 OR ME=喉部通气道 OR ME=喉罩呼吸支持 OR ME=喉部通气装置 OR ME=人工气道喉罩 OR ME=喉面罩 OR ME=喉面罩导气管 OR ME=气道管理)) AND ((ME=气管插管 OR ME=气管通气 OR ME=高级气道管理 OR ME=气道插管 OR ME=经口气管插管 OR ME=经鼻气管插管 OR ME=气管内插管 OR ME=快速气管插管 OR ME=直接喉镜插管 OR ME=气管内导管 OR ME=呼吸道插管 OR ME=人工气道建立 OR ME=机械通气插管 OR ME=气管导管插入 OR ME=气管内导管置入 OR ME=呼吸道插管 OR ME=插管法, 气管内 OR ME=插管法气管内 OR ME=插管术气管内)) AND (年份: 1989-01-01 TO 2025-05-31)

**The search strategy of Wanfang**

(主题:(急救医疗服务) or 主题:(院外) or 主题:(院前) or 主题:(现场急救) or 主题:(救护车上急救) or 主题:(现场医疗) or 主题:(移动医疗) or 主题:(入院前急救) or 主题:(急诊中心) or 主题:(急救医护) or 主题:(急救卫生保健服务) or 主题:(急诊医疗服务)) and (主题:(猝死, 心脏) or 主题:(心脏骤停) or 主题:(心搏骤停) or 主题:(心跳停止) or 主题:(心室颤动) or 主题:(心室停顿) or 主题:(心源性猝死) or 主题:(心脏停搏) or 主题:(心脏性猝死) or 主题:(心肺按压) or 主题:(心肺复苏) or 主题:(心脏停顿) or 主题:(无脉性电活动) or 主题:(无脉搏电活动) or 主题:(心脏猝死) or 主题:(心搏停止) or 主题:(心肺骤停) or 主题:(心跳骤停) or 主题:(心肺脑复苏) or 主题:(心脏骤停复苏) or 主题:(心脏病猝死) or 主题:(心性猝死) or 主题:(心室纤颤)) and (主题:(喉罩通气) or 主题:(喉罩) or 主题:(喉罩气道) or 主题:(LMA) or 主题:(喉罩通气道) or 主题:(喉部通气罩) or 主题:(喉部气道罩) or 主题:(喉部通气道) or 主题:(喉罩呼吸支持) or 主题:(喉部通气装置) or 主题:(人工气道喉罩) or 主题:(喉面罩) or 主题:(喉面罩导气管) or 主题:(气道管理)) and (主题:(气管插管) or 主题:(气管通气) or 主题:(高级气道管理) or 主题:(气道插管) or 主题:(经口气管插管) or 主题:(经鼻气管插管) or 主题:(气管内插管) or 主题:(快速气管插管) or 主题:(直接喉镜插管) or 主题:(气管内导管) or 主题:(呼吸道插管) or 主题:(人工气道建立) or 主题:(机械通气插管) or 主题:(气管导管插入) or 主题:(气管内导管置入) or 主题:(呼吸道插管) or 主题:(插管法, 气管内) or 主题:(插管法气管内) or 主题:(插管术气管内)) and (发表时间: 建库-20250531)

**The search strategy of PubMed**

(((((((((((((((((((((((((((Emergency Medical Services[Title/Abstract]) OR (Out of Hospital[Title/Abstract]) OR (Outside the hospital[Title/Abstract]) OR (emergency rescue medical service[Title/Abstract]) OR (Medical Service, Emergency[Title/Abstract]) OR (Service, Emergency Medical[Title/Abstract]) OR (Emergency Service, Medical[Title/Abstract]) OR (Medical Emergency Service[Title/Abstract]) OR (Services, Medical Emergency[Title/Abstract]) OR (Emergicenter[Title/Abstract]) OR (Prehospital Emergency Care[Title/Abstract]) OR (Emergency Care, Prehospital[Title/Abstract]) OR (Emergency Health Service[Title/Abstract]) OR (Health Service, Emergency[Title/Abstract]) OR (Service, Emergency Health[Title/Abstract]) OR (Emergency Care[Title/Abstract]) OR (Ambulances[Title/Abstract]) OR (First Aid[Title/Abstract]) OR (Before the hospital[Title/Abstract]) OR (On site first aid[Title/Abstract]) OR (First aid on ambulance[Title/Abstract]) OR (On site medical treatment[Title/Abstract]) OR (Mobile healthcare[Title/Abstract]) OR (First aid before admission[Title/Abstract]) OR (Emergency Center[Title/Abstract]) OR (Emergency medical staff[Title/Abstract]) OR (Emergency healthcare services[Title/Abstract]))) AND (((((((((((((((((((((((((((((((((asystole[Title/Abstract]) OR (cardiac arrest[Title/Abstract]) OR (heart arrest[Title/Abstract]) OR (heart stop pace[Title/Abstract]) OR (cardiogenic sudden death[Title/Abstract]) OR (cardiac ventricular fibrillation[Title/Abstract]) OR (ventricular fibrillation[Title/Abstract]) OR (Ventricular arrest[Title/Abstract]) OR (Sudden cardiac death[Title/Abstract]) OR (Arrest, Heart[Title/Abstract]) OR (Arrest, Cardiac[Title/Abstract]) OR (Cardiopulmonary Arrest[Title/Abstract]) OR (Arrest, Cardiopulmonary[Title/Abstract]) OR (Cardiopulmonary compressions[Title/Abstract]) OR (Cardiopulmonary Resuscitation[Title/Abstract]) OR (Non pulsatile electrical activity[Title/Abstract]) OR (No pulse electrical activity[Title/Abstract]) OR (Cardiac and pulmonary arrest[Title/Abstract]) OR (Cardiopulmonary cerebral resuscitation[Title/Abstract]) OR (OHCA[Title/Abstract]) OR (asystolia[Title/Abstract]) OR (asystoly[Title/Abstract]) OR (circulation arrest[Title/Abstract]) OR (circulatory arrest[Title/Abstract]) OR (heart asystole[Title/Abstract]) OR (heart standstill[Title/Abstract]) OR (CPR[Title/Abstract]) OR (Sudden Death, Cardiac[Title/Abstract]) OR (Cardiac Sudden Death[Title/Abstract]) OR (Cardiac Death, Sudden[Title/Abstract]) OR (Death, Cardiac Sudden[Title/Abstract]) OR (Death, Sudden Cardiac[Title/Abstract]) OR (Arrest, Sudden Cardiac[Title/Abstract])))) AND (((Ima-proseal[Title/Abstract]) OR (Laryngeal Mask Airway[Title/Abstract]) OR (LMA[Title/Abstract]) OR (Laryngeal Mask[Title/Abstract]) OR (Mask, Laryngeal[Title/Abstract]) OR (Airway, Laryngeal Mask[Title/Abstract]) OR (air-Q[Title/Abstract]) OR (air-Qsp[Title/Abstract]) OR (Aura-i[Title/Abstract]) OR (Aura40[Title/Abstract]) OR (AuraFlex[Title/Abstract]) OR (AuraGain[Title/Abstract]) OR (AuraOnce[Title/Abstract]) OR (AuraStraight[Title/Abstract]) OR (Classic Airway[Title/Abstract]) OR (CTrach[Title/Abstract]) OR (GuardianCPV[Title/Abstract]) OR (larynx mask[Title/Abstract]) OR (LarySeal[Title/Abstract]) OR (ProBreathe[Title/Abstract]) OR (ProSeal[Title/Abstract]) OR (Soft Seal[Title/Abstract]) OR (Unique Airway[Title/Abstract]))) AND ((((((((((((ETI[Title/Abstract]) OR (endotracheal intubation[Title/Abstract]) OR (Advanced airway management[Title/Abstract]) OR (tracheal cannula[Title/Abstract]) OR (Intratracheal Intubation[Title/Abstract]) OR (Intubation, Intratracheal[Title/Abstract]) OR (Intubation, Endotracheal[Title/Abstract]) OR (intubation, orotracheal[Title/Abstract]) OR (intubation, trachea[Title/Abstract]) OR (trachea intubation[Title/Abstract]) OR (orotracheal intubation[Title/Abstract]) OR (tracheal intubation[Title/Abstract])))) AND ("1900/01/01"[Date - Publication] : "2025/05/31"[Date - Publication])

**The search strategy of Web of science**

((TS=(Emergency Medical Services) OR TS=(Out of Hospital) OR TS=(Outside the hospital) OR TS=(emergency rescue medical service) OR TS=(Medical Service, Emergency) OR TS=(Service, Emergency Medical) OR TS=(Emergency Service, Medical) OR TS=(Medical Emergency Service) OR TS=(Services, Medical Emergency) OR TS=(Emergicenter) OR TS=(Prehospital Emergency Care) OR TS=(Emergency Care, Prehospital) OR TS=(Emergency Health Service) OR TS=(Health Service, Emergency) OR TS=(Service, Emergency Health) OR TS=(Emergency Care) OR TS=(Ambulances) OR TS=(First Aid) OR TS=(Before the hospital) OR TS=(On site first aid) OR TS=(First aid on ambulance) OR TS=(On site medical treatment) OR TS=(Mobile healthcare) OR TS=(First aid before admission) OR TS=(Emergency Center) OR TS=(Emergency medical staff) OR TS=(Emergency healthcare services)) AND (TS=(asystole) OR TS=(cardiac arrest) OR TS=(heart arrest) OR TS=(heart stop pace) OR TS=(cardiogenic sudden death) OR TS=(cardiac ventricular fibrillation) OR TS=(ventricular fibrillation) OR TS=(Ventricular arrest) OR TS=(Sudden cardiac death) OR TS=(Arrest, Heart) OR TS=(Arrest, Cardiac) OR TS=(Cardiopulmonary Arrest) OR TS=(Arrest, Cardiopulmonary) OR TS=(Cardiopulmonary compressions) OR TS=(Cardiopulmonary Resuscitation) OR TS=(Non pulsatile electrical activity) OR TS=(No pulse electrical activity) OR TS=(Cardiac and pulmonary arrest) OR TS=(Cardiopulmonary cerebral resuscitation) OR TS=(OHCA) OR TS=(asystolia) OR TS=(asystoly) OR TS=(circulation arrest) OR TS=(circulatory arrest) OR TS=(heart asystole) OR TS=(heart standstill) OR TS=(CPR) OR TS=(Sudden Death, Cardiac) OR TS=(Cardiac Sudden Death) OR TS=(Cardiac Death, Sudden) OR TS=(Death, Cardiac Sudden) OR TS=(Death, Sudden Cardiac) OR TS=(Arrest, Sudden Cardiac)) AND (TS=(Ima-proseal) OR TS=(Laryngeal Mask Airway) OR TS=(LMA) OR TS=(Laryngeal Mask) OR TS=(Mask, Laryngeal) OR TS=(Airway, Laryngeal Mask) OR TS=(air-Q) OR TS=(air-Qsp) OR TS=(Aura-i) OR TS=(Aura40) OR TS=(AuraFlex) OR TS=(AuraGain) OR TS=(AuraOnce) OR TS=(AuraStraight) OR TS=(Classic Airway) OR TS=(CTrach) OR TS=(GuardianCPV) OR TS=(larynx mask) OR TS=(LarySeal) OR TS=(ProBreathe) OR TS=(ProSeal) OR TS=(Soft Seal) OR TS=(Unique Airway)) AND (TS=(ETI) OR TS=(endotracheal intubation) OR TS=(Advanced airway management) OR TS=(tracheal cannula) OR TS=(Intratracheal Intubation) OR TS=(Intubation, Intratracheal) OR TS=(Intubation, Endotracheal) OR TS=(intubation, orotracheal) OR TS=(intubation, trachea) OR TS=(trachea intubation) OR TS=(orotracheal intubation) OR TS=(tracheal intubation))) NOT (SILOID==("PPRN")) AND PY=(1900-2025)

**The search strategy of Embase**

('emergency medical services':ab,ti OR 'out of hospital':ab,ti OR 'outside the hospital':ab,ti OR 'emergency rescue medical service':ab,ti OR 'medical service, emergency':ab,ti OR 'service, emergency medical':ab,ti OR 'emergency service, medical':ab,ti OR 'medical emergency service':ab,ti OR 'services, medical emergency':ab,ti OR emergicenter:ab,ti OR 'prehospital emergency care':ab,ti OR 'emergency care, prehospital':ab,ti OR 'emergency health service':ab,ti OR 'health service, emergency':ab,ti OR 'service, emergency health':ab,ti OR 'emergency care':ab,ti OR ambulances:ab,ti OR 'first aid':ab,ti OR 'before the hospital':ab,ti OR 'on site first aid':ab,ti OR 'first aid on ambulance':ab,ti OR 'on site medical treatment':ab,ti OR 'mobile healthcare':ab,ti OR 'first aid before admission':ab,ti OR 'emergency center':ab,ti OR 'emergency medical staff':ab,ti OR 'emergency healthcare services':ab,ti) AND (asystole:ab,ti OR 'cardiac arrest':ab,ti OR 'heart arrest':ab,ti OR 'heart stop pace':ab,ti OR 'cardiogenic sudden death':ab,ti OR 'cardiac ventricular fibrillation':ab,ti OR 'ventricular fibrillation':ab,ti OR 'ventricular arrest':ab,ti OR 'sudden cardiac death':ab,ti OR 'arrest, heart':ab,ti OR 'arrest, cardiac':ab,ti OR 'cardiopulmonary arrest':ab,ti OR 'arrest, cardiopulmonary':ab,ti OR 'cardiopulmonary compressions':ab,ti OR 'cardiopulmonary resuscitation':ab,ti OR 'non pulsatile electrical activity':ab,ti OR 'no pulse electrical activity':ab,ti OR (cardiac:ab,ti AND 'pulmonary arrest':ab,ti) OR 'cardiopulmonary cerebral resuscitation':ab,ti OR ohca:ab,ti OR asystolia:ab,ti OR asystoly:ab,ti OR 'circulation arrest':ab,ti OR 'circulatory arrest':ab,ti OR 'heart asystole':ab,ti OR 'heart standstill':ab,ti OR cpr:ab,ti OR 'sudden death, cardiac':ab,ti OR 'cardiac sudden death':ab,ti OR 'cardiac death, sudden':ab,ti OR 'death, cardiac sudden':ab,ti OR 'death, sudden cardiac':ab,ti OR 'arrest, sudden cardiac':ab,ti) AND ('ima proseal':ab,ti OR 'laryngeal mask airway':ab,ti OR lma:ab,ti OR 'laryngeal mask':ab,ti OR 'mask, laryngeal':ab,ti OR 'airway, laryngeal mask':ab,ti OR 'air q':ab,ti OR 'air qsp':ab,ti OR 'aura i':ab,ti OR aura40:ab,ti OR auraflex:ab,ti OR auragain:ab,ti OR auraonce:ab,ti OR aurastraight:ab,ti OR 'classic airway':ab,ti OR ctrach:ab,ti OR guardiancpv:ab,ti OR 'larynx mask':ab,ti OR laryseal:ab,ti OR probreathe:ab,ti OR proseal:ab,ti OR 'soft seal':ab,ti OR 'unique airway':ab,ti) AND (eti:ab,ti OR 'endotracheal intubation':ab,ti OR 'advanced airway management':ab,ti OR 'tracheal cannula':ab,ti OR 'intratracheal intubation':ab,ti OR 'intubation, intratracheal':ab,ti OR 'intubation, endotracheal':ab,ti OR 'intubation, orotracheal':ab,ti OR 'intubation, trachea':ab,ti OR 'trachea intubation':ab,ti OR 'orotracheal intubation':ab,ti OR 'tracheal intubation':ab,ti) AND ([1900-01-01]/sd NOT [2025-05-31]/ed)

**The search strategy of the Cochrane Library**

((Emergency Medical Services):ti,ab,kw OR (Out of Hospital):ti,ab,kw OR (Outside the hospital):ti,ab,kw OR (emergency rescue medical service):ti,ab,kw OR (Medical Service, Emergency):ti,ab,kw OR (Service, Emergency Medical):ti,ab,kw OR (Emergency Service, Medical):ti,ab,kw OR (Medical Emergency Service):ti,ab,kw OR (Services, Medical Emergency):ti,ab,kw OR (Emergicenter):ti,ab,kw OR (Prehospital Emergency Care):ti,ab,kw OR (Emergency Care, Prehospital):ti,ab,kw OR (Emergency Health Service):ti,ab,kw OR (Health Service, Emergency):ti,ab,kw OR (Service, Emergency Health):ti,ab,kw OR (Emergency Care):ti,ab,kw OR (Ambulances):ti,ab,kw OR (First Aid):ti,ab,kw OR (Before the hospital):ti,ab,kw OR (On site first aid):ti,ab,kw OR (First aid on ambulance):ti,ab,kw OR (On site medical treatment):ti,ab,kw OR (Mobile healthcare):ti,ab,kw OR (First aid before admission):ti,ab,kw OR (Emergency Center):ti,ab,kw OR (Emergency medical staff):ti,ab,kw OR (Emergency healthcare services):ti,ab,kw) AND ((asystole):ti,ab,kw OR (cardiac arrest):ti,ab,kw OR (heart arrest):ti,ab,kw OR (heart stop pace):ti,ab,kw OR (cardiogenic sudden death):ti,ab,kw OR (cardiac ventricular fibrillation):ti,ab,kw OR (ventricular fibrillation):ti,ab,kw OR (Ventricular arrest):ti,ab,kw OR (Sudden cardiac death):ti,ab,kw OR (Arrest, Heart):ti,ab,kw OR (Arrest, Cardiac):ti,ab,kw OR (Cardiopulmonary Arrest):ti,ab,kw OR (Arrest, Cardiopulmonary):ti,ab,kw OR (Cardiopulmonary compressions):ti,ab,kw OR (Cardiopulmonary Resuscitation):ti,ab,kw OR (Non pulsatile electrical activity):ti,ab,kw OR (No pulse electrical activity):ti,ab,kw OR (Cardiac and pulmonary arrest):ti,ab,kw OR (Cardiopulmonary cerebral resuscitation):ti,ab,kw OR (OHCA):ti,ab,kw OR (asystolia):ti,ab,kw OR (asystoly):ti,ab,kw OR (circulation arrest):ti,ab,kw OR (circulatory arrest):ti,ab,kw OR (heart asystole):ti,ab,kw OR (heart standstill):ti,ab,kw OR (CPR):ti,ab,kw OR (Sudden Death, Cardiac):ti,ab,kw OR (Cardiac Sudden Death):ti,ab,kw OR (Cardiac Death, Sudden):ti,ab,kw OR (Death, Cardiac Sudden):ti,ab,kw OR (Death, Sudden Cardiac):ti,ab,kw OR (Arrest, Sudden Cardiac):ti,ab,kw) AND ((Ima-proseal):ti,ab,kw OR (Laryngeal Mask Airway):ti,ab,kw OR (LMA):ti,ab,kw OR (Laryngeal Mask):ti,ab,kw OR (Mask, Laryngeal):ti,ab,kw OR (Airway, Laryngeal Mask):ti,ab,kw OR (air-Q):ti,ab,kw OR (air-Qsp):ti,ab,kw OR (Aura-i):ti,ab,kw OR (Aura40):ti,ab,kw OR (AuraFlex):ti,ab,kw OR (AuraGain):ti,ab,kw OR (AuraOnce):ti,ab,kw OR (AuraStraight):ti,ab,kw OR (Classic Airway):ti,ab,kw OR (CTrach):ti,ab,kw OR (GuardianCPV):ti,ab,kw OR (larynx mask):ti,ab,kw OR (LarySeal):ti,ab,kw OR (ProBreathe):ti,ab,kw OR (ProSeal):ti,ab,kw OR (Soft Seal):ti,ab,kw OR (Unique Airway):ti,ab,kw) AND ((ETI):ti,ab,kw OR (endotracheal intubation):ti,ab,kw OR (Advanced airway management):ti,ab,kw OR (tracheal cannula):ti,ab,kw OR (Intratracheal Intubation):ti,ab,kw OR (Intubation, Intratracheal):ti,ab,kw OR (Intubation, Endotracheal):ti,ab,kw OR (intubation, orotracheal):ti,ab,kw OR (intubation, trachea):ti,ab,kw OR (trachea intubation):ti,ab,kw OR (orotracheal intubation):ti,ab,kw OR (tracheal intubation):ti,ab,kw) AND (1900-2025).yr.
